# Downstream Pathways of Dystrophin Deficiency in Duchenne Muscular Dystrophy: Implications for Muscle Degeneration and Regeneration

**DOI:** 10.1002/jcsm.70333

**Published:** 2026-07-03

**Authors:** Raffaele Epis, Gabriele Rovetta, Giulia Ferrari, Chiara Bonfanti, Giorgia Careccia, Graziella Messina

**Affiliations:** ^1^ Department of Biosciences Università degli Studi di Milano Milan Italy

**Keywords:** anti‐inflammatory strategies, calcium homeostasis and mitochondrial function, cycles of degeneration and regeneration, Duchenne muscular dystrophy, myofibre stability and regeneration, oxidative stress, pharmacological treatments, satellite cell

## Abstract

**Background:**

Duchenne muscular dystrophy (DMD) is the most common and severe form of muscular dystrophy, primarily affecting skeletal muscle and leading to premature death. Although the loss of dystrophin has long been recognised as the primary cause of the disease, no definitive cure is currently available. As a consequence, therapeutic efforts have largely focused on mitigating disease progression rather than correcting the primary genetic defect. In this context, extensive research focuses on secondary pathological mechanisms, downstream cellular and molecular alterations triggered by dystrophin deficiency, that contribute to disease progression, particularly by affecting muscle degeneration and regeneration. While therapeutic strategies have traditionally aimed to enhance muscle regeneration, accumulating evidence indicates that limiting chronic degeneration and the associated degeneration–regeneration cycles may represent a more effective approach to preserve muscle integrity. Here, we examine the published evidence to delineate this shift in therapeutic perspective.

**Methods:**

This narrative review summarises preclinical and clinical strategies for DMD. We considered over 100 published articles, 85% from the last 15 years and analysing 40 different approaches, grouping them based on pathways targeted. For each study, therapeutic efficacy was assessed by focusing on the impact on promoting muscle regeneration versus limiting degeneration, based on morphological features, including muscle architecture, inflammation and fibrosis, as well as functional outcomes.

**Results:**

Our analysis revealed pathway‐specific benefits in skeletal muscle. Among calcium‐ and mitochondrial‐targeted interventions, 94.4% preserved muscle morphology and slowed down muscle regeneration. Myofibre stability approaches were evenly split, with 50% promoting regeneration and 50% delaying muscle wasting. Satellite cell–targeted therapies affecting proliferation and fusion enhanced muscle regeneration in most cases, while anti‐inflammatory strategies slowed muscle degeneration in 63.6% and promoted new myofibre formation in 36.4% of cases. Oxidative stress modulation preserved muscle structure in 85.7% of cases, while boosting muscle regeneration in the remaining therapies.

**Conclusions:**

Emerging evidence indicates a shift in DMD therapeutic focus, from strategies aimed primarily at enhancing muscle regeneration to approaches that limit repeated degeneration–regeneration cycles. Most current interventions act by modulating pathological processes that drive chronic muscle damage rather than by directly stimulating regeneration. Importantly, targeting multiple disease–relevant pathways within skeletal muscle has shown beneficial effects in preclinical models, supporting the concept that coordinated and temporally controlled modulation of key biological processes may represent an effective strategy to stabilise muscle tissue and delay disease progression.

## 1 Introduction

Muscular dystrophies (MDs) are a heterogeneous group of genetically inherited diseases with Duchenne muscular dystrophy (DMD) being the most common and severe form, with an incidence of 1:5000 male births, leading to premature death, typically in the patients' 30s [1]. DMD is an X‐linked genetic disorder, caused by mutations on the *DMD* gene, resulting in a dysfunctional form or absence of dystrophin [2]. Dystrophin is a structural protein that links the dystrophin‐associated protein complex (DAPC) to the actin in the cytoskeleton, in both skeletal myofibres and cardiomyocytes [2, 3], conferring stability against mechanical stress. Loss of dystrophin represents the primary pathogenic event in DMD, directly compromising sarcolemmal stability and increasing susceptibility to mechanical stress. This primary defect leads to muscle fibre damage and initiates recurrent cycles of degeneration and regeneration, ultimately driving progressive muscle wasting and fibrofatty infiltration. These processes are accompanied by the early activation of multiple downstream pathological mechanisms that act as major amplifiers of disease progression, including abnormal calcium influx, impaired calcium homeostasis, contraction‐induced damage, mitochondrial dysfunction, chronic inflammation and fibrosis. Together, these mutually reinforcing events exacerbate muscle degeneration and accelerate disease progression [4]. Higher intracellular Ca^2+^ levels strongly destabilise mitochondrial functionality that, in turn, actively participates in the degeneration of the tissue, also by exacerbating oxidative stress. Oxidative stress and inflammation are also known as secondary players to promote muscle wasting. Indeed, if their action is crucial for tissue regeneration after the initial damage, their constant presence leads to further degeneration, ROS production, immune cell response and scar deposition [5]. In parallel, the dysfunction of satellite cells (SCs), the resident muscle stem cells, is recognised as an active contributor to DMD [6]. Indeed, in dystrophic muscles, SCs cannot adequately replace damaged myofibres due to both intrinsic and extrinsic factors. In addition, even if myogenesis is not completely blocked, the newly formed myofibres still possess the same genetic mutation in the *DMD* gene, resulting in the generation of fragile myofibres whose destiny is to degenerate once they face mechanical stress. Therefore, besides the genetic cause of DMD, different secondary players actually participate in the recurring degeneration/regeneration cycles characterising skeletal muscle affected by MD.

While the ideal treatment for DMD would involve the restoration of a functional dystrophin, all clinical trials conducted so far have proven limited effectiveness in patients [7, 8, 9]. Several factors contribute to this lack of success, including the challenge of targeting all skeletal muscles throughout the body; the limited ability of therapeutic cells to cross vessels, resulting in poor distribution and engraftment across muscle tissue; the reduced survival and regenerative potential of transplanted cells within the hostile dystrophic environment; the large size of the *Dystrophin* gene, which exceeds the carrying capacity of most viral vectors; the immune response against the viral vectors commonly used to deliver dystrophin and/or against dystrophin, which may be perceived as a non‐self‐protein; and the substitution of myofibres with fibrotic scar that render skeletal muscle unresponsive to any therapeutic treatment.

Although a definitive cure remains elusive, conservative interventions targeting the secondary hallmarks of DMD can significantly improve patient survival and quality of life and therefore remain critically important. This review focuses on secondary therapeutic strategies that target downstream pathological mechanisms arising from dystrophin loss. Rather than categorising therapies solely according to their molecular targets, we analyse them based on their functional impact on muscle tissue, distinguishing between approaches aimed at accelerating the regeneration and those primarily limiting chronic degeneration. Within this framework, we highlight an emerging conceptual shift from strategies designed to promote regeneration toward interventions that stabilise myofibres and slow disease progression.

Accordingly, we examine the main conservative therapeutic strategies developed to date to determine whether their efficacy derives primarily from promoting regeneration or from limiting degeneration–regeneration cycles.

## 2 Modulating Calcium Homeostasis and Mitochondrial Function to Alleviate the DMD Phenotype

**FIGURE 1:**
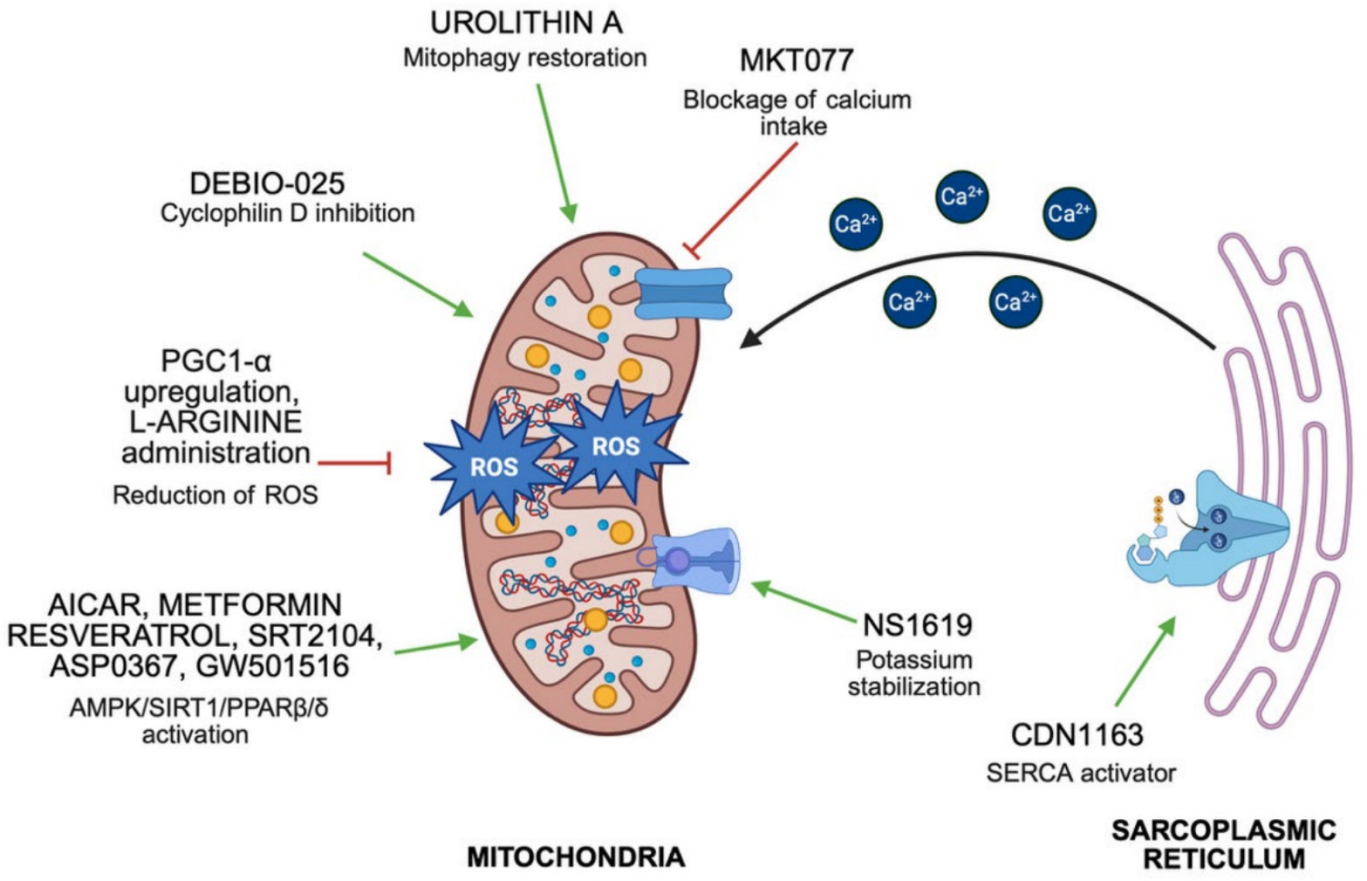
Therapeutic interventions towards calcium and mitochondria in MDs. CDN1163 enhances Ca^2+^ reuptake into the sarcoplasmic reticulum, while MKT077 limits mitochondrial Ca^2+^ entry. Debio‐025 preserves mitochondrial integrity by inhibiting Cyclophilin D, and NS1619 supports K^+^ homeostasis. Urolithin A restores mitophagy, and activation of PGC1α, AMPK, SIRT1 and PPARβ/δ reduces ROS and improves mitochondrial function.

An important consequence of dystrophin loss at the myofibre level is the destabilisation of sarcolemmal integrity, which results in dysregulated Ca^2+^ influx and abnormally elevated cytosolic calcium concentrations [10]. Ca^2+^ overload contributes to mitochondrial dysfunction, including mitochondrial swelling, loss of membrane potential and impaired energy production, ultimately promoting myofibre damage and cell death. Accordingly, substantial efforts have been directed toward restoring Ca^2+^ homeostasis and preserving mitochondrial function (Figure 1; Tables 1 and 2). Most of these approaches act by limiting skeletal muscle degeneration, supporting the view that calcium dysregulation represents a key downstream trigger that amplifies disease progression in DMD.

**TABLE 1:** Summary of study characteristics and main results in preclinical models.

.References	Treatment	Target	Preclinical mice model	Age	Dosage	Administration	Study period	Muscular output
Goonasekera et al. (2011)	Genetic approach	SERCA1 upregulation	*Mdx* mice and *Sgcd*‐/‐ mice	12 weeks old	NA	NA	NA	Decreased fibrosis, decreased serum CK, decreased EBD incorporation, decreased centrally nucleated myofibres
Nogami et al. (2021)	CDN1163	SERCA1 upregulation	*Sgcd*‐/‐ mice	14 weeks old	40 mg/kg	IP, daily	7 weeks	Decreased fibrosis, decreased serum CK, decreased degenerating myofibres and centrally nucleated myofibres, decreased fibrosis, increased force by grip test
Millay et al. (2008)	Genetic approach	Cyclophilin D inhibition	*Scgd−/−* mice	6 weeks old	NA	NA	NA	Decreased EBD incorporation, decreased necrosis, decreased centrally nucleated myofibres, increased CSA, increased force
Millay et al. (2008)	Debio‐025	Cyclophilin D inhibition	*Mdx* mice	4 weeks old	50 mg/kg	SC, twice a day	6 weeks	Reduced mitochondria swelling, decreased fibrosis, decreased centrally nucleated myofibres
Wissing et al. (2010)	Debio‐025	Cyclophilin D inhibition	*Mdx* mice	6 weeks old	50–80 mg/kg	SC or oral, daily	6 weeks	Decreased centrally nucleated myofibers, increased CSA, decreased fibrosis, decreased inflammatory cells infiltration, increased grip strengths, decreased serum CK
Dubinin et al. (2021)	Alisporivir (Debio‐025)	Cyclophilin D inhibition	*Mdx* mice	8 weeks old	5 mg/kg	IP, daily	4 weeks	Increased muscle function by wire hang test, decreased serum AST, increased calcium retention capacity
Dubinin et al. (2024)	MKT077	GRP75 inhibition	*Mdx* mice	8 weeks old	5 mg/kg	IP, daily	4 weeks	Increased calcium retention capacity, decreased centrally nucleated myofibres, decreased fibrosis, increased CSA, decreased serum CK, increased grip strength
Dubinin et al. (2022)	NS1619	MitoBKCa inhibition	*Mdx* mice	8 weeks old	0.5 mg/kg	IP, daily	4 weeks	Increased potassium efflux and calcium retention, decreased centrally nucleated myofibres, decreased fibrosis
Chan et al. (2014)	Genetic approach	PGC1‐α upregulation	*Mdx* mice and *mdx*/*utrn*‐/‐ mice	5 weeks old	NA	NA	NA	Decreased serum CK, decreased centrally nucleated myofibers, increased CSA, decreased EBD incorporation, increased muscle function by treadmill
Gonzalez‐Sanchez et al. (2018)	Obestatin	PGC1‐α upregulation	*Mdx* mice	8 weeks old	500 nmol/kg	IM, every 3 days	4 weeks	Increased force, decreased centrally nucleated myofibers, decreased serum CK, decreased CSA, increased SDH staining, decreased IgG+ myofibres
Ljubicic et al. (2011); Pauly et al. (2012)	AICAR	AMPK upregulation	*Mdx* mice	6 weeks old	500 mg/kg	SC or IP, daily	4 weeks	Increased mitochondrial COX activity and Utrophin levels, increased sarcolemmal structural integrity, decreased centrally nucleated myofibres, increased slow myofibres
Jahnke et al. (2012)	AICAR	AMPK upregulation	*Mdx* mice	12 weeks old	250 mg/kg	Oral, daily	4 weeks	Increased muscle weight, increased muscle force, increased NADH activity, increased centrally nucleated myofibres, decreased IgM, decreased fibrosis
Juban et al. (2018)	Metformin	AMPK upregulation	*Mdx* mice	8–10 weeks old	200 mg/kg/day	Beverage	3.5 weeks	Decreased fibrosis and necrosis, decreased pro‐inflammatory markers, increased anti‐inflammatory markers, increased CSA, decreased Pax7+ nuclei, increased force
Chalkiadaki et al. (2014)	Genetic approach	SIRT1 upregulation	*Mdx* mice	8–10 weeks old	NA	NA	NA	Decreased degeneration, decreased EBD incorporation, increased muscle function by treadmill, decreased serum CK, increased Utrophin and PGC1α levels
Giovarelli et al. (2025)	SRT2104	SIRT1 upregulation	DMD Drosophila M.; Mdx mice	8 weeks old	100 mg/kg/day	Diet	12 weeks	Increased muscle function by treadmill test, decreased necrosis, fibrosis and inflammatory cells infiltration, increased regenerating myofibres and MYOZ1 area
Ljubicic et al. (2014)	Resveratrol	SIRT1‐PGC1‐α upregulation	*Mdx* mice	6–7–weeks old	100–500 mg/kg/day	Diet	6–12 weeks	Increased COX IV activity, increased slow and fast 2a myofibres, increased Utrophin expression
Sebori et al. (2018)	Resveratrol	SIRT1‐PGC1‐α upregulation	*Mdx* mice	9 weeks old	40–400–4000 mg/kg/day	Diet	56 weeks	Decreased centrally nucleated myofibres, increased CSA, decreased serum CK, increased muscle function by wire hanging test and rotarod
Miura et al. (2009)	GW501516	PPARβ/δ upregulation	*Mdx* mice	5–7 weeks old	25 mg/kg	Oral, daily	4 weeks	Decreased degenerating myofibres, increased slow and fast 2a myofibres, decreased EBD incorporation
Jahnke et al. (2012)	GW501516	PPARβ/δ upregulation	*Mdx* mice	12 weeks old	7.5 mg/kg	Oral, daily	4 weeks	Increased muscle weight, increased muscle force, increased NADH activity, increased centrally nucleated myofibres, decreased IgM, fibrosis and inflammation
Hnia et al. (2008)	L‐arginine	L‐arginine upregulation	*Mdx* mice	5 weeks old	200 mg/kg	IP, daily	2 weeks	Increased centrally nucleated myofibres, decreased non‐muscle area, decreased pro‐inflammatory cytokines
Luan et al. (2021)	Urolithin A	Mitophagy upregulation	*Mdx* mice	3 weeks old	50 mg/kg/day	Diet	10 weeks	Decreased inflammation, increased CSA, decreased EBD incorporation, decreased serum CK, decreased centrally nucleated myofibres, decreased fibrosis, increased muscle function by treadmill
Tinsley et al. (1998)	Genetic approach	Utrophin upregulation	*Mdx* mice	10–12 weeks old	NA	NA	NA	Increased muscle force, decreased degenerating and centrally nucleated myofibres
Tinsley et al. (2011)	Ezutromid (SMT C1100)	Utrophin upregulation	*Mdx* mice	3 weeks old	50 mg/kg	IP, daily	4 weeks	Decreased serum CK, decreased force drop, decreased centrally nucleated myofibres, increased muscle force
Wu et al. (2025)	MyoAAV‐UA	Utrophin upregulation	*Mdx* mice	2 weeks old	5 × 10^3^ vg/kg	IV	6 months	Decreased centrally nucleated myofibres, decreased fibrosis, decreased serum CK
Russel et al. (2023)	EDG‐5506	Fast myosin ATPase inhibition	*Mdx* mice; DMD dogs	4–5 weeks old	3 mg/kg	Oral, daily	3–8–12 weeks	Decreased degeneration, decreased EBD incorporation, increased muscle force, decreased fibrosis, decreased centrally nucleated myofibres
Bonato et al. (2023)	Genetic approach	Cyclin D3 ablation	*Mdx* mice	8–10 weeks old	NA	NA	NA	Increased SDH staining, decreased degeneration, decreased inflammatory cell infiltration, decreased necrosis, decreased regenerating myofibres, decreased centrally nucleated myofibres, increased CSA, increased muscle function by treadmill test
Reyes et al. (2015)	Genetic approach	Fnip1 ablation	*Mdx* mice	8–12 weeks old	NA	NA	NA	Decreased serum CK, decreased centrally nucleated myofibres
Blanchet et al. (2012)	Genetic approach	E2F1 ablation	*Mdx* mice	8 weeks old	NA	NA	NA	Decreased centrally nucleated myofibres, decreased inflammatory infiltration, decreased EBD incorporation, decreased serum CK, increased muscle function and force
Rossi et al. (2017)	Genetic approach	NFIX ablation	*Sgca‐/‐* mice; *Mdx* mice	3–5–8–12–24 weeks old	NA	NA	NA	Decreased EBD incorporation, decreased inflammatory cells infiltration, increased regenerating myofibres, increased muscle function by treadmill test, increased SDH staining, decreased centrally nucleated myofibres, increased CSA, decreased fibrosis
Colussi et al. (2008)	HDAC2 siRNA	HDAC2 inhibition	*Mdx* mice	8 weeks old	NA	NA	NA	Increased CSA, decreased inflammatory cell infiltration, increased muscle function by treadmill test
Consalvi et al. (2013)	Givinostat	pan‐HDAC inhibition	*Mdx* mice	6 weeks old	1–5–10 mg/kg	Oral, daily	15 weeks	Increased CSA, decreased fibrosis and fat deposition, decreased inflammation, increased muscle function by treadmill test, decreased EBD incorporation
Osseni et al. (2022)	Tubastatin A	HDAC6 inhibition	*Mdx* mice	7 weeks old	25 mg/kg	IP, daily	4 weeks	Increased muscle force, increased CSA, decreased centrally nucleated myofibres, decreased fibrosis
Bogdanovich et al. (2002)	Mstn Blocking Antibodies	Mstn inhibition	*Mdx* mice	4 weeks old	60 mg/kg	IP, weekly	12 weeks	Increased muscle mass and CSA, increased muscle force, increased centrally nucleated myofibres, increased single fiber area, decreased serum CK
Iskenderian et al. (2018)	Follistatin	Mstn inhibition	*Mdx* mice	3 weeks old	3–10–30 mg/kg	SC, 2 times a week	12 weeks	Increased CSA, increased muscle force, decreased necrosis and fibrosis, decreased inflammatory cells infiltration
St. Andre et al. (2017)	Domagrozumab	Mstn inhibition	*Mdx* mice	8 weeks to 1 year old	10 mg/kg	IP, weekly	8 weeks	Increased muscle weight, increased muscle force, increased CSA
St. Andre et al. (2017)	Domagrozumab	Mstn inhibition	*Nonhuman primates*	3–5 years old	30 mg/kg	IV, weekly	8 weeks	Increased muscle volume
Gregorevic et al. (2004)	IGF1	IGF‐1 upregulation	*Mdx* mice	6 weeks old	1 mg/kg	Osmotic pump, daily	8 weeks	Increased muscle force, increased SDH staining, increased CSA
Schertzer et al. (2006)	Recombinant human IGF‐I	IGF‐1 upregulation	*Mdx* mice	20–23 weeks old	1.5 mg/kg	Osmotic pump, daily	8 weeks	Increased SDH staining, switch toward oxidative fiber metabolism, decreased contraction‐induced damage
Alexander et al. (2014)	miR‐486 genetic upregulation	PTEN inhibition	*Mdx* mice	8–16 weeks old	NA	NA	NA	Decreased centrally nucleated myofibres, increased CSA, decreased EBD incorporation, decreased serum CK, increased muscle function by treadmill test, increased muscle force
Yue et al. (2021)	Genetic approach	PTEN inhibition	*Mdx* mice	8–9 weeks old	NA	NA	NA	Increased muscle weight, increased CSA, increased muscle force, decreased necrosis, fibrosis and inflammatory infiltrate, decreased EBD incorporation, decreased serum CK
Yue et al. (2021)	VO‐Ohpic	PTEN inhibition	*Mdx* mice	3 weeks old	10 mg/ml	IP, daily	3 weeks	Increased muscle force, increased muscle function by treadmill test, decreased necrosis, decreased regenerating myofibres
Dorchies et al. (2013)	Tamoxifen	Estrogen receptor inhibition	*Mdx* mice	3 weeks old	100 mg/kg	Diet	63 weeks	Increased muscle function, decreased serum CK, decreased fibrosis, increased centrally nucleated myofibres
Petrany et al. (2020)	Genetic approach	Mymk inhibition in myofibers	*Mdx* mice	8 weeks old	NA	NA	28 weeks	Decreased serum CK, decreased necrosis and fibrosis, decreased EBD incorporation, increased muscle force
Fontelonga et al. (2019)	Sunitinib	α7β1 integrin upregulation	*Mdx* mice	4 weeks old	1 mg/kg	Oral, 3 times a week	8 weeks	Increased muscle force, increased regenerating myofibres, increased centrally nucleated myofibres, increased CSA, increased Pax7+ cells
von Maltzahn et al. (2012)	Genetic approach	Wnt7a upregulation	*Mdx* mice	10 weeks old	NA	NA	NA	Increased muscle weight, increased muscle force, increased slow MyHC+ myofibres, decreased IgG+ myofibres, decreased centrally nucleated myofibres, increased Pax7+ cells
Matias‐Valiente et al. (2024)	Isolecanoric acid	GSK3β inhibition	*Mdx* mice	12 weeks old	100 mg/kg	IP, daily	6 days	Decreased fibrosis
Taglietti et al. (2023); Cojocaru et al. (2025)	Forskolin	Adenylyl cyclase upregulation	DMD rats	4 weeks old	2.5 mg/kg	IP, twice a week	12 weeks	Increased muscle force, decreased fibrosis, increased regenerating myofibres, increased Pax7+ cells
De Luca et al. (2005)	Cyclosporine A	Calcineurin inhibition	*Mdx* mice	4 to 5 weeks old	10 mg/kg	Diet	4–8 weeks	Increased mouse force, decreased degenerating myofibres, decreased fibrosis
Liang et al. (2018)	Cenicriviroc	Dual CCR2/CCR5 chemokine receptor antagonist	*Mdx* mice	2 weeks old	20 mg/kg	IP, daily	4 weeks	Decreased inflammatory cells infiltration, decreased number of regenerating myofibres, increased muscle force
Dubuisson et al. (2022)	MCC950	NLRP3 inhibition	*Mdx* mice	4 weeks old	40 mg/kg/day for 4 weeks, then 80 mg/kg/day for 4 weeks	Beverage	8 weeks	Increased muscle function in wire test and grip test, decreased inflammation and oxidative stress, increased slow and fast 2a myofibres, decreased fibrosis
Dort et al. (2021)	Resolvin‐D2	Gpr18 agonist	*Mdx* mice and *mdx*/u*trn*‐/‐ mice	10 weeks and 6 weeks old	5 μg/kg	IP, daily	3 weeks	Decreased inflammation, increased MyoG+ cells, decreased fibrosis, increased muscle function in hang test, increased muscle force
Dort et al. (2023)	PSB‐KD107	Gpr18 agonist	*Mdx* mice	10 weeks old	1 mg/kg	IP, weekly	3 weeks	Decreased inflammation, increased MyoG+ cells, decreased fibrosis, increased muscle force
Saclier et al. (2022)	Genetic approach	NFIX silencing in myeloid cells	*Sgca‐/‐ mice*	8–16–24 weeks old	NA	NA	NA	Increased CSA, decreased EBD incorporation, decreased fibrosis, decreased inflammatory cells infiltration, increased FAPs apoptosis
Sali et al. (2012)	Prednisone	Glucocorticoid receptor activation	*Mdx* mice	8–10 weeks old	1 mg/kg SC; 5 mg/kg Oral	SC or oral, daily	25 weeks	Decreased body weight, increased muscle function, decreased inflammation, decreased centrally nucleated myofibres
Wehling‐Henricks et al. (2004)	Prednisone	Glucocorticoid receptor activation	*Mdx* mice	2 weeks old	0.75 mg/kg	IP, 5 times a week	2 weeks	Decreased inflammatory cell infiltration, decreased IgG+ myofibres, decreased centrally nucleated myofibres
Heier et al. (2013)	VBP15	Glucocorticoid Receptor activation	*Mdx* mice	2 or 6 weeks old	5–15–30–45 mg/kg	Oral, daily	6–16 weeks	Increased muscle force, decreased inflammation, decreased fibrosis
Sun et al. (2025)	Aminoguanidine hemisulfate	ROS attenuation	*Mdx* mice	6 weeks old	40 mg/kg	IP, daily	2 weeks	Decreased regenerating myofibres, increased muscle force, increased expression of Pax7, Myf5, MyoD, decreased MyoG expression
Saclier et al. (2020)	Cyanidin	ROS attenuation	*Sgca*‐/‐ mice	3 weeks old	NA	Diet	5–25 weeks	Decreased inflammatory cells infiltration, increased muscle function by treadmill, switch toward oxidative fiber metabolism, decreased fibrosis
Angelini et al. (2024)	Cyanidin	ROS attenuation	*Sgca*‐/‐ mice, mdx mice	3 weeks old	7.7 mg/mL	Beverage	5 weeks	Reduction of side effects induced by Trametinib treatment comprising decreased necrosis, fibrosis and calcifications
Sitzia et al. (2015)	Mix of dietary natural polyphenols (ProAbe)	ROS and inflammation attenuation	Wild‐type and *mdx* mice	3 months old	NA	Diet	4 weeks	Decreased necrotic and regenerating myofibres, decreased fibrosis, increased muscle function by treadmill
Segatto et al. (2020)	JQ1	BRD4 inhibition	*Mdx* mice	10 weeks old	20 mg/kg	IP, daily	2 weeks	Decreased necrotic and regenerating myofibres, decreased inflammation and fibrosis, increased muscle function by treadmill, inverted screen test and wire test
Careccia et al. (2021)	3S‐HMGB1	HMGB1 redox isoforms balance	*Mdx* and *Sgca*‐/‐ mice	8 weeks old	500 μg	IP, twice a week	3 to 6 weeks	Increased centrally nucleated myofibres and satellite cells number, decreased necrosis, decreased inflammatory infiltrate and fibrosis, increased muscle function by treadmill and inverted hanging tests
Cervia et al. (2024)	Plumbagin	Inflammation and oxidative stress reduction	*Mdx* mice	4 weeks old	250 mg/kg/day	Diet	3 months	Decreased necrosis, decreased fibrosis, increased muscle function
Coles et al. (2024)	Benfotiamine	Oxidative stress attenuation	*Mdx* mice	4 weeks old	10 mg/kg/day	Diet	2 weeks	Decreased necrosis and inflammation, decreased centrally nucleated myofibres, increased muscle function by grip test

Abbreviations: AMPK, AMP‐activated protein kinase; AST, aspartate aminotransferase; BRD4, bromodomain‐containing protein 4; CCR2, C‐C chemokine receptor type 2; CCR5, C‐C chemokine receptor type 5; CK, creatine kinase; COX, Cyclooxygenase; CSA, cross‐sectional area; E2F1, E2 promoter‐binding factor 1; EBD, Evans Blue Dye; FAPs, fibro‐adipogenic progenitors; Fnip1, Folliculin interacting protein 1; Gpr18, G‐protein‐coupled receptor 18; GRP75, glucose‐regulated protein 75; GSK3β, glycogen synthase kinase‐3 beta; HDAC, histone deacetylase; HDAC2, histone deacetylase 2; HDAC6, histone deacetylase 6; HMGB1, high‐mobility group box 1; IGF‐1, insulin‐like growth factor 1; IP, intraperitoneal injection; Mstn, myostatin; Myf5, myogenic factor 5; MyHC, myosin heavy chain; Myh1, myosin heavy chain 1; Myh7, myosin heavy chain 7; Mymk, myomaker; MyoD, myoblast determination protein; MyoG, myogenin; MYOZ1, myozenin 1; NA, not applicable; NFIX, nuclear factor 1 X; NLRP3, nucleotide‐binding domain, leucine‐rich–containing family, pyrin domain–containing‐3; Pax7, paired box gene 7; PGC1α, peroxisome proliferator–activated receptor γ coactivator 1; PPARβ/δ, peroxisome proliferator–activated receptors; ROS, reactive oxygen species; SC, subcutaneous injection; SDH, succinate dehydrogenase; SERCA1, sarcoplasmic reticulum calcium ATPase 1; SIRT1, NAD‐dependent type III deacetylase; Wnt7a, Wnt family member 7A.

**TABLE 2:** Summary of study characteristics and main results in clinical trials.

References	Compound/drug	Target	Age	Intervention and dosage	Administration route	Study period	Participants	Frequency of supplementation	Outcomes
Hafner et al. (2019)	L‐citrulline + Metformin	Mitochondrial metabolism (AMPK and nNOS) upregulation	8.2 years (± 1.1)	L‐citrulline 2.5 g per dose and metformin 250 mg per dose	L‐Citrulline (via powder in sachets dissolved in water); 250 mg of metformin (via metformin hydrochloride tablets)	26 weeks	47 ambulant DMD patients	Three times daily	No significant differences in timed function and muscle force tests, proximal and axial, and distal motor function. Motor function measure first dimension subscore decrease of 5.5%
Kawamura et al. (2020)	Resveratrol (3,5,4′‐trihydroxy‐trans‐stilbene)	SIRT1 upregulation	DMD: 21.0 (± 9.7); BMD: 33.5 (± 8.3); FCMD: 14.5 (± 2.5) years	Initial dose of 500 mg/day (8 weeks); dose was then increased to 1000 and finally to 1500 mg/day	Oral administration	24 weeks	5 DMD; 4 BMD; 2 FCMD	Daily	Mean motor function measure scores increased significantly from 34.6 to 38.4; twofold increase in the mean quantitative muscle testing scores of scapula elevation and shoulder abduction; mean CK levels decreased considerably by 34%; diarrhoea and abdominal pain
Ito et al. (2022)	ASP0367	PPARδ upregulation	18 to 55 years	1 mg ASP0367 or matching placebo	Oral administration	Day 1; Day 14	64 (single‐dose cohort) and 37 (multiple‐dose cohort)	Single dose or multiple dose	No clinically significant changes observed on laboratory or electrocardiographic evaluation. Treatment‐ and dose‐dependent upregulation of six PPARδ target genes
Liu et al. (2022)	Urolithin A	Mitophagy upregulation	Placebo: 71.0 (± 4.58); Urolithin A: 72.5 (± 5.24) years	1000 mg urolithin A or placebo for 4 months.	Oral administration	26 weeks	Urolithin A (n = 33); placebo (*n* = 33)	Daily	Improvements in the 6‐min walk distance and maximal ATP production not significant. Long‐term urolithin A supplementation beneficial for muscle endurance and plasma biomarkers
Donovan et al. (2025)	Sevasemten (EDG‐5506)	Fast myosin ATPase inhibition	Healthy volunteers: single dose 35.0 (± 9.7), or multidose 35.8 ± (± 9.3); BMD: 33.0 (± 6.2) years	Single oral suspension up to 135 mg	Oral administration	15 months and 13 days	97 Healthy volunteers in seven single‐dose cohorts (*N* = 57) and five multiple‐dose cohorts (*N* = 40)	Single ascending dose or multiple ascending dose	Average maximal reductions of 70% for CK, 98% for fast skeletal muscle Troponin I and 45% for myoglobin
Mercuri et al. (2024)	Givinostat (EPIDYS)	HDAC inhibition	Givinostat: 9.8 (8.1–11.0); placebo: 9.9 (8.3–11.4)	Regimen 1: Initial dose of 20‐70 mg; Regimen 2: Initial dose of 13–47 mg	Oral administration	72 weeks	Givinostat (*n* = 118); placebo (*n* = 61). 170 (95%) completed the study.	Twice a day	Four‐stair climb assessment worsened in both groups over the study period. Smaller decline with Givinostat than placebo. The dose of Givinostat was reduced after an interim safety analysis, but no new safety signals were reported
Kirschner et al. (2010)	Ciclosporin A + Prednisone	Calcineurin inhibition	Placebo: 7.0 (±1.4); Ciclosporin A: 7.1 (±1.5)	Dose of 3.5–4.0 mg/kg bodyweight	Capsules (oral solution for patients unable to swallow the capsules)	12 months	Placebo (*n* = 73); Ciclosporin A (n = 73)	Ciclosporin A or placebo (3 months, twice daily) + intermittent Prednisone (10 days). Combined treatment continued for 12 months.	No improvement in muscle strength or functional abilities, but safe and well tolerated
Wagner et al. (2008)	MYO‐029	Myostatin inhibition	Placebo: 39.3 (± 13.3); MYO‐029 1 mg/kg: 37.2 (± 9.5); MYO‐029 3 mg/kg: 37.1 (± 13.6); MYO‐029 10 mg/kg: 40.2 (± 11.5); MYO‐029 30 mg/kg: 44.3 (± 10.2)	Cohort 1 at 1 mg/kg; Cohort 2 at 3 mg/kg; Cohort 3 at 10 mg/kg; Cohort 4 at 30 mg/kg	Intravenous administration	9 months	Placebo (*n* = 29); 1 mg/kg (*n* = 27); 3 mg/kg (*n* = 27); 10 mg/kg (*n* = 27); 30 mg/kg (*n* = 6)	Every 2 weeks for 6 months	Good safety and tolerability. No improvements noted in exploratory end points of muscle strength or function
Conklin et al. (2018)	Vamorolone	GR activation	4 to 7 years	Vamorolone 0.25, 0.75, 2.0, and 6.0 mg/kg/day for 2 weeks (2‐week follow‐up off)	Oral administration	4 weeks	48 boys with DMD	Daily	Improved safety of Vamorolone versus glucocorticoids, beneficial changes in bone turnover, reduction in adrenal suppression, anti‐inflammatory mechanism of action and a beneficial effect on plasma membrane stability (reduced CK)
Finkel et al. (2021)	Edasalonexent (CAT‐1004)	NF‐κB inhibition	Edasalonexent: 5.65 (± 1.048); Placebo: 5.77 (± 0.995)	Three doses of 33 mg/kg each	Softgel capsules orally administered with food containing at least 8 g of fat	52 weeks	131 patients: Edasalonexent (*n* = 88), Placebo (*n* = 43)	Three divided doses of approximately 33mg/kg each	North Star Ambulatory Assessment not statistically significant. Well‐tolerated treatment and mild adverse events

Abbreviations: AMPK, AMP‐activated protein kinase; ATP, adenosine triphosphate; BMD, Becker muscular dystrophy; CK, creatine kinase; DMD, Duchenne muscular dystrophy; FCMD, Fukuyama congenital muscular dystrophy; GR, glucocorticoid receptor; HDAC, histone deacetylase; NF‐κB, nuclear factor kappa‐light‐chain‐enhancer of activated B cells; nNOS, neuronal nitric oxide synthase; PPARβ/δ, peroxisome proliferator‐activated receptors; QMT, quantitative muscle testing; SIRT1, NAD‐dependent type III deacetylase.

One critical target tested is sarcoplasmic reticulum calcium ATPase 1 (SERCA1), a Ca^2+^ pump responsible for Ca^2+^ reuptake into the sarcoplasmic reticulum during muscle relaxation [11], found to have reduced activity in DMD. The transgenic overexpression of SERCA1 in mouse models of MDs resulted in histological mitigation of the disease, comprising decreased percentage of Evans Blue Dye (EBD) positive fibres, centrally nucleated myofibres (CNM), regenerating myofibres and fibrosis [12]. Similarly, pharmacological treatment with the allosteric SERCA activator, CDN1163, ameliorated the phenotype of the murine DMD model, the *mdx* mouse, with reduced necrosis and fibrosis due to decreased degeneration/regeneration cycles, pointing to SERCA1 activators as new promising conservative therapy in DMD [13]. Alternatively, other approaches directly target mitochondrial Ca^2+^ overload. Of note, Cyclophilin D, encoded by the *Ppif* gene, is a mitochondrial matrix isomerase that directly regulates Ca^2+^ and reactive oxygen species (ROS)–dependent mitochondrial permeability, which directly trigger cellular necrosis. Genetic ablation of *Ppif* or pharmacological inhibition by Debio‐025 reduced disease progression by slowing down cycles of degeneration and regeneration. Particularly, Debio‐025 reduced mitochondrial swelling, improved histological examination, with decreased number of EBD+ myofibres and central nucleated myofibres and ameliorated muscle functionality [14, 15]. However, Debio‐025 treatment induced suppression of mitochondrial biogenesis and altered the dynamics of organelles, inhibiting mitochondria fission and fusion, indicating some negative aspects of such therapy [16] and lightening the needs for further investigation. On the same line, rhodacyanine MKT077, an inhibitor of glucose‐regulated protein 75 (GRP75)‐mediated Ca^2+^ transfer to mitochondria, improved mitochondrial ultrastructure with decreased central nucleation and fibrosis. Therefore, mitochondrial overload of Ca^2+^ was revealed to be a primary driver of DMD pathology and its inhibition delays degeneration/regeneration cycles [17]. Besides, mitochondrial dysfunction is also accompanied by a reduction in K^+^ transport. NS1619, an inhibitor of mitochondrial large‐conductance calcium‐activated potassium channel (mitoBK_Ca_), was tested in *mdx* mice and resulted in normalisation of channel expression and K^+^ levels, increased Ca^2+^ mitochondrial retention capacity, amelioration of mitochondrial structure and mitigation of oxidative stress. This effect induced a reduction of fibrosis and degeneration/regeneration loops (decreased percentage of CNM) and normalised the sarcomere size [18]. All these therapeutic approaches targeting calcium or potassium overload mitigated the dystrophic phenotype by decreasing degeneration and regeneration cycles.

Impairments in mitochondrial biogenesis and dynamics have been described in DMD pathogenesis, highlighting the complexity of targeting mitochondrial pathways while also supporting the therapeutic potential of strategies that promote mitochondrial biogenesis and oxidative metabolism. Peroxisome proliferator‐activated receptor γ coactivator 1 (PGC1) is known to transcriptionally regulate and activate several factors, controlling mitochondrial biogenesis and functions, neuromuscular junction genes expression and muscle fibre type switching [19, 20]. Interestingly, PGC1‐α is highly expressed in muscles enriched in oxidative fibres such as the soleus, which are more protected by DMD progression compared with glycolytic fibres [21] and characterised by higher mitochondrial content and oxidative rate. Genetic PGC1α overexpression [22] or indirect stimulation via Obestatin [23] improved *mdx* phenotype by delaying degeneration and regeneration cycles. Further efforts converging on PGC1α activation were developed by modulating key sensors that monitor the cell's metabolic state and substrate availability: AMP‐activated protein kinase (AMPK), nicotinamide adenine dinucleotide (NAD+)–dependent type III deacetylase (SIRT1) and peroxisome proliferator–activated receptors (PPARs), deeply studied and investigated in MD [24]. AMPK regulates PGC1‐α to initiate mitochondrial biogenesis in response to increased intramuscular AMP:ATP ratio [25]. The importance of AMPK activation to attenuate dystrophic phenotype was demonstrated by genetic loss or gain‐of‐function models. In particular, AMPK activation, achieved by mutation in one subunit, caused a shift toward slower myosin heavy chain isoforms, increased PGC1‐α expression and mitochondrial synthesis [26]. Pharmacological administration of AICAR, AMPK activator 5‐aminoimidazole‐4‐carboxamide‐1‐β‐d‐ribofuranoside, enhanced AMPK activation and upregulated markers of slow, oxidative myogenic programme, inducing a switch toward more oxidative metabolism in glycolytic muscles and leading to decreased IgM staining and delayed degeneration/regeneration cycles [27]. On the same line, a new approach to activate AMPK was developed involving the use of a bioactive AMPK agonist, named MK‐8722. A single dose of MK‐8722 significantly activated AMPK, which in turn led to upregulation of PGC1‐α and resulted in lower myogenic programme activation [28]. Additionally, upregulation SIRT1, [29] through SRT2104 [30] or Resveratrol [31, 32], improved mitochondrial gene expression, reduced muscle necrosis and enhanced muscle performance. However, SRT2104 and Resveratrol appear to differentially influence the cycles of muscle degeneration and regeneration, with SRT2104 reported to promote regenerative processes, whereas Resveratrol to decrease degeneration/regeneration cycles. Those differences may be caused by the cellular targeting of the two drugs, particularly SRT2104 may favour SC activation and muscle repair, while Resveratrol may primarily stabilise muscle fibres through its antioxidant and anti‐inflammatory properties, thereby reducing the need for repeated regeneration cycles. This suggests that SIRT1 activation may exert context‐dependent effects on muscle homeostasis depending on the specific pharmacological modulator and its downstream signalling profile. Resveratrol supplementation was further tested in a clinical trial in patients with different forms of MD, such as Duchenne, Becker or Fukuyama [33]. Twenty‐four weeks of treatment of patients showed improvements in motor function, reduced CK levels and had few mild side effects, as diarrhoea and abdominal pain [33]. On the same line, GW501516, a peroxisome proliferator‐activated receptors (PPARβ/δ) selective agonist, improved mitochondrial activity and promoted the switch from glycolytic to oxidative metabolism, accompanied by decreased area of regeneration and degeneration [34, 35]. GW501516 was also tested in a clinical trial but was dismissed due to tumorigenic effects in long‐term studies in mice and rats [36]. Recently, other compounds to stabilise PPARβ/δ were tested and ASP0367 showed a good safety profile in adults [37].

Another way to target mitochondrial function involved supplementation with L‐arginine [38], which enhanced mitochondrial respiration, decreased ‘non‐muscle area’, intended as the area between the myofibres (defined as peripheral nuclei fibres or internal nuclei fibres) and increased the number of CNM. Metformin [39], an antidiabetic drug, indirectly increased AMPK activity, which in turn enhanced nNOS signalling, leading to increased myofibres' cross‐sectional area (CSA), reduced muscle damage and fibrosis, reprogramming macrophage toward an anti‐inflammatory phenotype. A first pilot trial in DMD patients demonstrated that the combined L‐arginine/Metformin treatment prevented loss of motor function and muscle degeneration [40]. Subsequently, a randomised, placebo‐controlled trial showed good tolerability, but the reduction in motor function decline was detectable only in a subgroup of patients [41].

Finally, restoring mitophagy, the autophagic removal of damaged mitochondria, offers therapeutic potential. The treatment with Urolithin A, a mitophagy activator, rescued mitophagy in DMD worms, mice and primary myoblasts derived from patients, paving the rational for ongoing clinical trials [42]. In dystrophic mice, Urolithin A treatment improved muscle morphology and muscle function by attenuating degeneration/regeneration cycles, as evidenced by reduced necrosis, decreased expression of later markers of myogenic cells' differentiation and lower number of CNM. Beyound DMD models, Urolithin A has been tested in an aged population with reduced mitochondrial function and exercise capacity. Although no significant improvements were observed in the 6‐min walk test or maximal ATP production, long‐term supplementation enhanced muscle endurance and plasma biomarkers [43], suggesting its potential as a therapeutic candidate for DMD.

In summary, therapeutic strategies targeting Ca^2+^ homeostasis, mitochondrial function and metabolic regulators converge on a central principle: mitigating DMD pathology by stabilising myofibres and slowing degeneration. Approaches such as SERCA1 activation, inhibition of mitochondrial Ca^2+^ overload and modulation of PGC1‐α, AMPK, SIRT1 and PPAR pathways reduce EBD positive myofibres, decrease the proportion of CNM and limit fibrofatty infiltration. These interventions preserve myofibre integrity and mitochondrial health while tempering pathological cycles of degeneration and regeneration. Importantly, reducing degeneration does not compromise regenerative capacity. Persistent mitochondrial dysfunction impairs muscle repair, whereas restored mitochondrial health fosters effective myogenic regeneration. Thus, fewer CNMs reflect diminished pathological regeneration, while improved mitochondrial function creates a supportive environment for productive regeneration when needed. Collectively, these findings highlight the dual therapeutic benefit of attenuating degeneration while promoting efficient, coordinated muscle regeneration in DMD.

## 3 Balancing Myofibre Stability, Metabolism and Growth to Counteract DMD Progression

**FIGURE 2:**
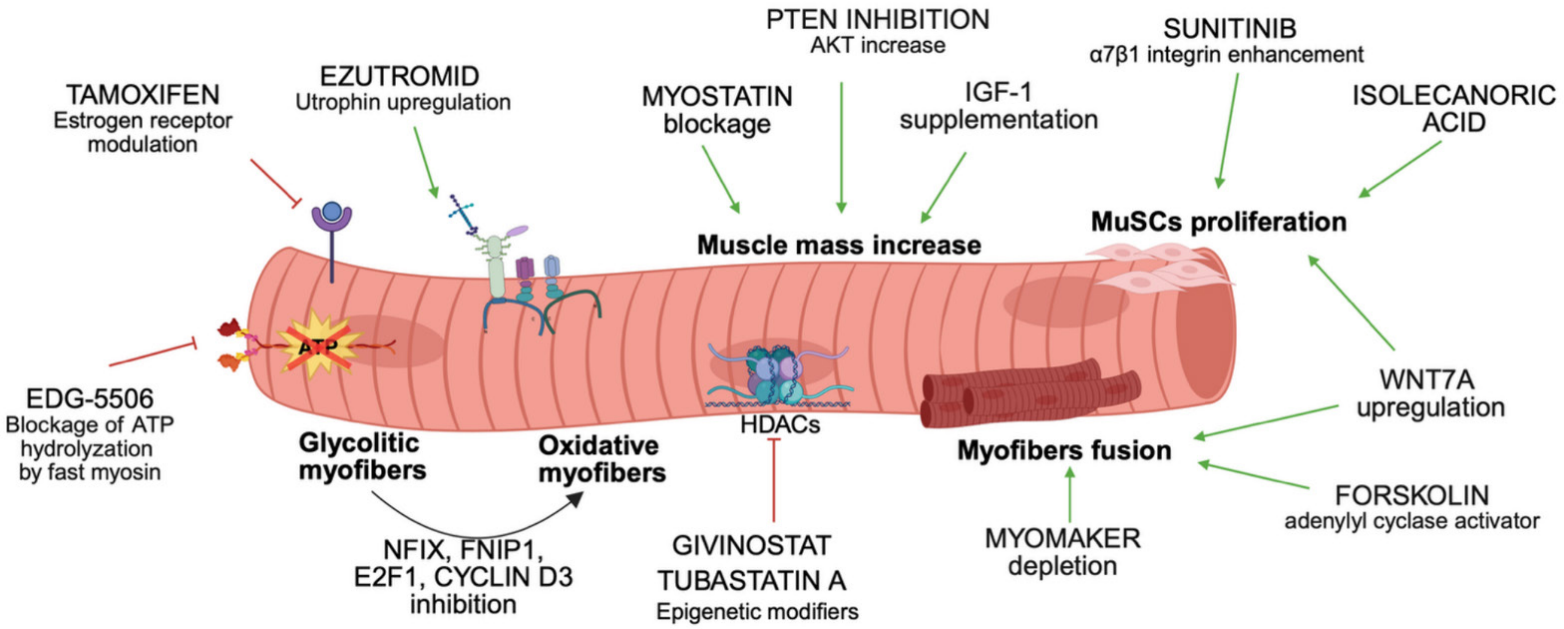
Myofibers and satellite cell interventions to slow down MD degeneration. Stabilisation of myofibers can be achieved through utrophin upregulation, fast myosin inhibition (EDG‐5506) or modulation of oestrogen receptors (Tamoxifen). Muscle mass and regeneration are enhanced by myostatin or PTEN inhibition and IGF‐1 supplementation, while inhibition of NFIX, FNIP1, E2F1 and Cyclin D3 slows degeneration. Epigenetic modifiers such as Givinostat and Tubastatin A further support muscle preservation. Satellite cell activity is promoted by Sunitinib, isolecanoric acid, Wnt7a upregulation and Forskolin, whereas *Myomaker* depletion enhances myofibre fusion.

Dystrophic skeletal muscle degeneration is primarily driven by myofibres' instability during contraction and relaxation in the absence of dystrophin. Targeting myofibres' stability to prevent these cycles is a promising therapeutic avenue (Figure 2; Tables 1 and 2). Historically, one strategy to mitigate the DMD phenotype has involved stabilising myofibres and delaying degeneration by upregulating utrophin (UTRN), a homologue of dystrophin [43]. In *mdx* mice, genetic overexpression of UTRN or pharmacological overexpression through SMT C1100 delayed the degeneration/regeneration cycles highlighted by decreased central nucleated myofibres in diaphragm, EDL and soleus, mitigating the dystrophic course of pathology, underlined by decreased creatine kinase (CK) levels in serum, a well‐established diagnostic marker for muscle degeneration and improving functional tests like endurance and forelimb force [44]. Over the years, a clinical trial was tested in patients, but the development of the promising Ezutromid (SMT C1100) was stopped after a Phase 2, as the amelioration seen halfway was not maintained throughout the duration of the trial. Further analyses indicated that Ezutromid's pharmacokinetic properties likely contributed to the lack of sustained efficacy. The compound undergoes extensive metabolism in humans, leading to reduced active drug exposure over time [45]. This highlights the importance of achieving durable target expression and adequate muscle exposure in utrophin‐based approaches. The promising conservative approach through the upregulation of UTRN expression is still at the forefront of research. Indeed, MyoAAV‐UA were recently generated and proved efficacy in mitigating *mdx* mice phenotype, supporting ongoing investigation into this strategy [46].

Another class of innovative approaches stands by the knowledge that fast myosin isoforms are more susceptible to contraction‐induced damage compared with the slow ones [21]. Indeed, EDG‐5506, a selective inhibitor of fast myosin ATPase, could mitigate muscle injury in *mdx* mice and DMD dogs by delaying cycles of degeneration and regeneration [47]. A Phase I, double‐blind trial of EDG‐5506 had been conducted in healthy volunteers and adults with Becker MD (BMD), a milder form of DMD characterised by partially functional or reduced levels of dystrophin. No serious adverse events were reported, and encouraging results were obtained in BMD patients. Indeed, a reduction of CK serum level, concomitant with a reduction of 125 proteins, signature of BMD, was lowered with treatment [48]. Currently, two different Phase 2 EDG‐5506 clinical trials are ongoing for Becker and Duchenne MDs patients, which are expected to end by 2026 (https://clinicaltrials.gov/study/NCT05540860, https://clinicaltrials.gov/study/NCT05291091).

As alternative, different approaches aim to change myofibre metabolism. Genetic ablation in *mdx* mice of cyclin D3 [49], a cell cycle‐regulatory protein or of Folliculin interacting protein 1 (Fnip1) [50], modulator of mTORC1 function, was shown to drive a slow‐twitch program, delaying degeneration and regeneration. In the last decades, new targets entered preclinical evaluation, among which the inhibition of transcription factors resulted promising but challenging due to lack of druggable sites [51, 52]. *Mdx* mice genetically depleted for E2 promoter‐binding Factor 1 [53], E2F1, a transcription factor regulating cell cycle and DNA synthesis, converted myofibre metabolism towards an oxidative one, improved mitochondrial activity and muscle performance, finally delaying muscle degeneration [54]. Another conservative approach involves the inhibition of the transcription factor nuclear factor 1 X (NFIX). NFIX ablation in dystrophic mice models induced a significant decrease in macrophage infiltration (F4/80 staining) and Collagen 1 deposition, leading to a rescue of the functional abilities in the treadmill test. Furthermore, SDH staining revealed a switch toward a more oxidative phenotype after NFIX ablation, accompanied by a delay in degeneration/regeneration cycles. Similar results were also obtained in adult dystrophic mice through electroporation of a specific plasmid containing a short hairpin against NFIX, suggesting the possibility to mitigate dystrophy even when the disease already arose [55]. Conversely, once the pharmacological repurposing of a MEK inhibitor, named Trametinib, was tested to inhibit NFIX in dystrophic animals, off‐target effects not due to NFIX inhibition induced the appearance of ectopic calcification [56, 57]. However, the specific targeting of NFIX remains fundamental to mitigate the dystrophic phenotype.

Another effective way to treat human disease is the correction of pathogenic patterns. Histone deacetylase (HDAC) inhibitors can modulate the enzymes responsible for histone deacetylation, thereby influencing the regulation of gene expression [58]. Class I HDAC expression was found to be altered in *mdx* skeletal muscle compared with controls, with HDAC2 levels statistically increased. Hence, silencing of HDAC2 by siRNA mitigated the dystrophic phenotype, resulting in improved muscle performance. Further, HDAC2 silencing led to an increased fusion index in vitro and CSA in mice, suggesting that the phenotypic amelioration is mediated through the stimulation of the muscle regeneration program [59]. Among different HDAC inhibitors, Givinostat (ITF2357) was already in Phase I safety study in children with juvenile arthritis [60], and therefore, it was investigated in *mdx* mice. Givinostat treatment in *mdx* animals reduced necrosis (EBD+ areas), increased myofibres size, reduced inflammation (MPO staining), fibrosis (calculated on Masson trichrome staining) and ameliorated fatigue resistance of animals in the treadmill test [61]. Preclinical studies set the ground for the evaluation of Givinostat in DMD patients: 20 ambulant DMD patients, at early stage of diseases, were enrolled and treated for, at least, 12 months with Givinostat [62]. The drug was safe and well tolerated, and the treatment increased the fraction of muscle tissue in the biopsies and reduced the fibrotic deposition, proving that Givinostat administration counteracted histological disease progression. Subsequently, functional evaluation of Givinostat on DMD patients was conducted in a multicentre, randomised, double‐blind, placebo‐controlled, Phase 3 trial. Results of the four‐stair climb assessment worsened in both the placebo and treated group over the period; however, the decline was significantly smaller with Givinostat than with placebo [63]. On March 21, 2024, Duvyzat (Givinostat) was approved by Food and Drug Administration (FDA) as oral medication for the treatment of DMD in patients older than six. Conversely, Tubastatin A, an HDAC6 inhibitor, improved pathological outcome by increasing utrophin levels, reducing the number of CNM and fibrotic area, resulting in decreased degeneration/regeneration loops, higher muscle fibre integrity and increased grip strength [64].

Lastly, other approaches targeting myofibres focus on factors promoting muscle growth. Among them, the blockage of myostatin (Mstn) [65], a myokine that negatively regulates muscle growth, has been largely investigated [66]. Mstn inhibition through different strategies (monoclonal antibodies, modified follistatin, Mstn inhibitory prodomain) resulted to be beneficial in *mdx* mice [67, 68, 69]. However, domagrozumab, an anti‐Mstn antibody, failed to translate into clinical benefit, leading Pfizer to interrupt its drug development [70, 71, 72]. Nonetheless, it is important to define the potential contributors to this unsuccessful translation to clinic outcome, comprising Mstn level in mice versus human, confounding effects due to corticosteroid treatment, increase in type II fibres and decrease in mitochondria number as reported in the work of Amthor and colleagues [73]. On the same line, insulin‐like growth factor 1 (IGF‐1) administration [74, 75] or phosphatase and tensin homologue (PTEN) inhibition [76] proved to promote AKT activity and to stimulate anabolism over catabolism. The two approaches resulted in amelioration of *mdx* phenotype by acting differentially on degeneration and regeneration cycles. IGF‐1 administration accelerated myogenesis and regeneration [77, 78], while PTEN inhibition, by miR‐486 overexpression, decreased the number of CNM, decelerating cycles of degeneration and regeneration [79]. This discrepancy may be explained by the distinct biological roles of IGF‐1 and PTEN in skeletal muscle, as well as by the broader effects resulting from miR‐486 overexpression. Indeed, IGF‐1 is a pleiotropic growth factor that not only stimulates SC proliferation and differentiation but also acts directly on myoblasts and myotubes to promote myogenic differentiation and hypertrophy through activation of PI3K/Akt signalling pathways, which is essential for both repair and growth of muscle fibres [80]. In contrast, PTEN functions as a key negative regulator of the PI3K/Akt pathway and also contributes to the regulation of metabolic signalling and structural stability in mature myofibres [81]. In SCs, PTEN plays an important role in maintaining the quiescent stem cell pool, whereas its loss promotes premature activation and differentiation, potentially leading to depletion of the stem cell reservoir and long‐term impairment of muscle regenerative capacity [82]. However, Alexander et al. reported a reduction in degeneration cycles following PTEN modulation suggesting that additional cellular targets or compensatory mechanisms may contribute to the overall phenotype, possibly mitigating excessive SC activation. Together, these distinct cellular targets and functions help explain why modulation of IGF‐1 versus PTEN may lead to different effects on muscle regeneration outcomes. Also, the repurposing of the anticancer drug Tamoxifen, an oestrogen receptor modulator, resulted to mitigate dystrophic phenotype enhancing skeletal muscle regeneration [83].

In conclusion, therapeutic strategies targeting myofibre stability, metabolism and growth aim conversely to delay cycles of degeneration and regeneration or contrarily push regeneration. Several approaches, such as utrophin upregulation, selective inhibition of fast myosin, NFIX inhibition and metabolic reprogramming preserve myofibre integrity, lowering CSA, CNM and fibrosis. By stabilising myofibres, these strategies slow down degeneration/regeneration cycles ultimately improving muscle function. NFIX inhibition represents a clear example of this paradigm, as it slows degeneration/regeneration cycles while concomitantly delaying myoblast fusion and regenerative processes. Despite this attenuation of regeneration, overall morphological and functional improvements are observed. On the contrary, interventions like Givinostat, IGF‐1 administration or Mstn inhibition accelerate regeneration increasing CSA and CNM. Of note, inhibition of HDACs through Givinostat resulted to be beneficial in clinic. Collectively, these findings highlight a shift in paradigm of therapeutic strategies in DMD: Historically focused mainly on enhancing regeneration, current approaches also emphasise the importance of slowing degeneration/regeneration cycles while fine‐tuning regenerative responses to achieve sustained functional benefit.

## 4 Satellite Cells‐Based Strategies to Restore Muscle Function in DMD

SC dysfunctionality is a crucial aspect that exacerbates muscle wasting in MDs. Different studies associated changes in muscle stem cells' ability to differentiate and efficiently replenish the quiescent pool to both intrinsic and extrinsic causes [6]. This dysfunction limits the capacity for effective muscle regeneration and contributes to disease and has been the focus of therapeutic investigations for many years (Figure 2; Table 1).

Myomaker (Mymk), a transmembrane protein essential for myoblast fusion, exemplifies the central role of SCs in muscle regeneration [84]. Genetic depletion of Mymk in SCs of 2‐month‐old *mdx* mice led to a severe dystrophic phenotype characterised by increased fibrosis, inflammation and decreased grip strength and muscle weight. Notably, this phenotype closely resembled what was observed following inducible ablation of the entire SC population using tamoxifen treatment in RosaDTA/+ Pax7‐ires‐CreERT2 mice, suggesting that the regenerative contribution of SCs in dystrophic muscle relies predominantly on their fusogenic capacity, which is critically dependent on Mymk expression [85]. In contrast, other genetic models such as Mapk3^−/−^ Mapk1^fl/fl‐Pax7Cre‐ER^ or Pax7^Cre‐ERT2^ Rosa26‐DTA mice have reported a mitigation of dystrophic phenotype in both *mdx* and *Sgcd−/−* mouse models [86]. The discrepancy likely reflects differences in the timing and dosage of tamoxifen administration, as well as the functional state of SCs at the time of depletion. While loss of Mymk specifically impairs myoblast fusion while preserving the presence of SCs, DTA‐mediated ablation eliminates SCs in a Pax7‐dependent manner, thereby preventing their participation in ongoing regenerative cycles. Additionally, Mymk deletion in mature myofibres showed beneficial effects: reduced necrosis, fibrosis and CK levels and improved muscle function. These findings highlight a context‐dependent role of Mymk, essential during myoblast fusion for regeneration and detrimental for the membrane integrity of mature myofibres [85]. Collectively, these findings highlight that both SC function and Mymk activity exert distinct and stage‐specific effects in MD. Efficient myoblast fusion is essential for regeneration, but in chronic conditions, incomplete or dysregulated regenerative attempts, rather than the mere presence or absence of SCs, may drive disease progression.

Similarly, enhancing α7β1 integrin via Sunitinib treatment increased expression of myogenic markers, improved muscle strength and boosted SC proliferation. Sunitinib‐treated *mdx* mice revealed an enhanced expression of developmental myosin heavy chain (dMyHC) fibres, a reduction in muscle damage and an increase in the number of CNM, indicating that Sunitinib treatment promotes regeneration of muscle in vivo [87]. Also, Wnt7a overexpression promoted muscle regeneration, switched myofibres towards type I via Mef2C and activated the AKT/mTOR pathway, expanding the SC population, with functional and histological improvements in *mdx* muscles [88].

Natural and pharmacological agents have also shown promise. Isolecanoric acid, a GSK3β inhibitor, enhanced SC proliferation and regeneration through Wnt/β‐catenin modulation [89]. Forskolin administration, by activating adenylyl cyclase, increased fusion index and boosted regeneration in rats, resulting in decreased fibrotic area and improved running performances, but the long‐term use raised concerns regarding efficacy and cardiac safety [90, 91]. Particularly, intraperitoneal injection of Forskolin induced the worsening of histopathological index in DMD rats, accompanied by developed defects in cardiac conduction, evidenced by an increase in the QTpc interval compared with WT control.

Those studies indicate that disease outcome in MD depends not only on enhancing SC–mediated regeneration but also critically on its timing and regulation. While most approaches aim to stimulate SC differentiation, excessive regeneration may be detrimental by promoting exhaustion of the SC pool. Delayed regeneration could be protective by preserving the SC pool, while insufficient repair may instead promote fibrotic repair. The divergent effects observed across models indicate that dysregulated regeneration, rather than SC depletion alone, drives disease progression, highlighting the need for therapeutic strategies that precisely control the timing of muscle regeneration.

## 5 Anti‐Inflammatory Strategies to Counteract DMD

**FIGURE 3:**
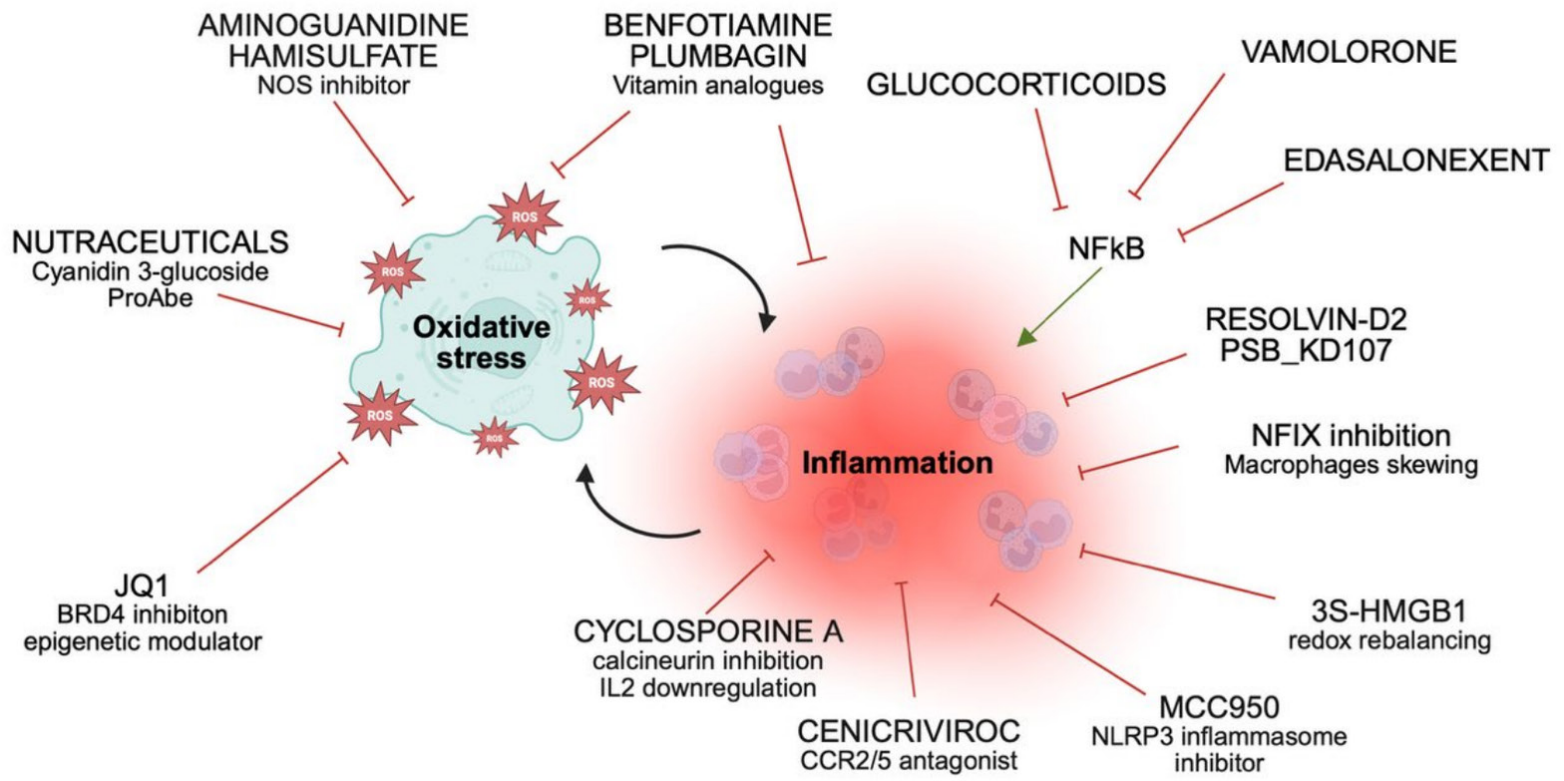
Therapeutic strategies against oxidative stress and inflammation in muscular dystrophies. Chronic oxidative stress and inflammation drive DMD progression. NFκB inhibition by glucocorticoids, Vamorolone and Edasalonexent reduces inflammation, while agents such as 3S‐HMGB1, Cyclosporine A, Cenicriviroc, MCC950, Resolvin‐D2 and PSB‐KD107 further limit immune cell infiltration. Oxidative stress is counteracted by compounds including aminoguanidine hemisulfate, JQ1, Benfotiamine, Plumbagin and nutraceuticals. Some interventions act on both pathways, highlighting their interconnected role in dystrophic pathology.

Among the extrinsic factors influencing SCs' behaviour, inflammation plays a central role. Inflammation is also a hallmark of DMD, considered beneficial in clearing necrotic tissue and to trigger initial phases of regeneration, but was found to be deleterious during the chronic condition, significantly contributing to disease progression. The precise targeting of inflammation (Figure 3; Tables 1 and 2) is crucial both to mitigate muscle degeneration but also to enable successful regeneration.

Growing number of therapeutic strategies have developed to interfere with key proinflammatory pathways, including those involved in macrophage recruitment, cytokine signalling and inflammasome activation. Cyclosporine A, a neutral lipophilic cyclic undecapeptide, widely used for allograft rejection and graft‐versus‐host disease, showed immunosuppressive activity and was tested in dystrophic mice models. Cyclosporine A inhibits Ca^2+^/calmodulin‐dependent phosphatase Calcineurin, and in turn promotes the downregulation of Interleukin 2, essential for T cells' activation and proliferation [92]. Cyclosporine A treatment in *mdx* mice displayed a reduction in muscle degeneration, as well as in the number of CNM. The histological amelioration was also followed by decreased CK levels in blood and diminished fibrotic accumulation [93]. However, in a randomised controlled trial, Cyclosporine A resulted to be safe and well tolerated but treatment had no effect on muscle functionality [94]. Similarly, Cenicriviroc, a dual chemokine receptor (CCR2 and CCR5) antagonist, already under development for HIV treatment [95] as well as in a phase 2b trial of nonalcoholic steatohepatitis patients [96], mitigated disease progression in *mdx* mice delaying degeneration/regeneration cycles [97]. Another approach tested was MCC950, inhibitor of NLRP3 inflammasome complex, which diminished the percentage of necrotic area and partially rescued muscle force and resistance to fatigue [98]. Even if the number of CNM did not change in treated muscles, the expression of *Myh3*, a marker of early muscle regeneration, was decreased by treatment, suggesting the slowing down of the degeneration/regeneration processes [98]. Besides, Resolvin‐D2, a bioactive lipid derived from omega‐3 fatty acids, was shown to switch macrophage toward anti‐inflammatory phenotype, which is known to promote myoblasts' differentiation and fusion, and stimulate myogenesis without impacting on SCs' proliferation [99]. In line, PSB‐KD107, a synthetic agonist of the Resolvin‐D2 receptor Gpr18, was developed as a potential treatment for MDs. The administration of PSB‐KD107 to *mdx* mice resulted in reduced inflammation, promoted an increase in the number of regenerating myofibres, reduced the fibrotic deposition and improved muscle functionality [100].

The contribution of inflammation to the dystrophic phenotype results from the complex coexistence of pro‐inflammatory macrophages and cytokines alongside anti‐inflammatory macrophages and their associated factors within the muscle. Both cell populations play key roles in disease progression and targeting either has been shown to provide therapeutic benefit. In macrophage populations, transcription factor NFIX is preferentially expressed by anti‐inflammatory macrophages and is critically involved in promoting the phenotypic transition from pro‐inflammatory macrophages toward an anti‐inflammatory state [101]. Hence, the selective ablation of NFIX in macrophages mitigated the dystrophic phenotype by decreasing necrosis and percentage of CNM, improving muscle strength in the grip test. Dystrophic macrophages lacking NFIX expressed more TNFα and less TGFβ1, promoting apoptosis of fibro‐adipogenic progenitors (FAPs), delaying fibrotic deposition and muscle wasting [102]. Therefore, NFIX ablation in macrophages ameliorates DMD phenotype by decelerating degeneration/regeneration processes.

Beyond the targeting of pathways that selectively regulate immune cell responses, particular interest has been directed toward signalling pathways that are shared by immune cells and myogenic cells. Modulation of these common pathways exerts direct effects on both cell types, simultaneously shaping the inflammatory milieu and intrinsically regulating muscle cell behaviour, including differentiation and regeneration. In this context, NF‐κB represents a central inflammatory regulator and has been reported to be aberrantly upregulated in dystrophic muscles [103]. Specifically, the targeted depletion of IKKβ in the myeloid cell lineage led to inhibition of NF‐κB, resulting in reduced inflammation and necrosis in dystrophic muscles, demonstrating that sustained inflammation contributes to degeneration [104]. Notably, the specific deletion of IKKβ in myofibres promoted regeneration, supporting that IKKβ‐mediated NF‐κB activation inhibits the regeneration process. Therefore, inhibition of NF‐κB pathway affects differentially cell populations, decreasing inflammation by myeloid cells, and promoting regeneration of myogenic cells. Glucocorticoids represent the standard care for DMD, mitigating inflammation and prolonging survival. These steroid hormones bind the glucocorticoids receptor (GR) and suppress proinflammatory signals by upregulating IKBα and GILZ and by directly inhibiting NF‐κB and AP‐1 transcriptional activity [105, 106]. This leads to reduced expression of IL‐6, TNFα and COX‐2 and increased levels of Annexin A1 and IL‐10 [107]. Prednisone, a synthetic glucocorticoid, has shown clinical benefit in improving motor function and slowing down weakness progression [108, 109, 110, 111]. However, the modulation of pathways shared by multiple cell types may also lead to unintended effects that must be carefully considered. Indeed, prolonged high‐dose glucocorticoid treatment can impair muscle function by promoting the expression of atrophy‐related genes, inducing insulin resistance and reducing protein synthesis [112]. Therefore, the selective targeting of specific cell populations is of fundamental importance. Vamorolone, a dissociative steroid, retains anti‐inflammatory properties without GR‐mediated side effects, showing functional benefits in preclinical and clinical studies, which led to its approval for DMD [113, 114]. On October 26, 2023, the FDA approved AGAMREE (Vamorolone) for the treatment of DMD in patients aged 2 years and older. In December 2023, the EMA granted marketing authorisation for AGAMREE for the treatment of DMD in patients aged 4 years and older, regardless of their underlying mutation or ambulatory status. Similarly, Edasalonexent, a dual NF‐κB inhibitor, was well tolerated and showed a trend toward slowing disease progression in younger paediatric DMD patients, although it did not meet its primary efficacy endpoints and further development was discontinued [115].

All considered, inflammation is essential for initiating tissue repair and regeneration following muscle injury, but chronic and unresolved inflammation contributes significantly to muscle degeneration, fibrosis and muscle stem cell exhaustion. Therapeutic strategies such as Cyclosporine A or NFIX ablation reduce the number of CNM and fibrotic deposition, preserving muscle integrity through distinct modulation of macrophage populations. Cyclosporine A favours a shift toward an anti‐inflammatory macrophage profile, whereas NFIX ablation alters macrophage signalling in a manner that limits fibrotic remodelling. On the contrary, anti‐inflammatory interventions that increase the number of CNM and CSA, such as Resolvin‐D2, promote muscle regeneration. Importantly, interventions that decelerate degeneration–regeneration cycles may preserve muscle integrity over time, whereas indiscriminate suppression or activation of inflammatory signalling risks impairing effective regeneration. Collectively, these findings indicate that precise control of the inflammatory milieu is required not only to mitigate muscle degeneration but also to preserve SC regenerative capacity, enabling regeneration to occur more efficiently and durably over time.

## 6 Targeting Oxidative Stress to Mitigate DMD Progression

DMD is recognised as a chronic disease, in which oxidative stress and inflammation are deeply linked and mutually reinforcing. Early in disease progression, oxidative stress arises from mitochondrial dysfunction, leading to the generation of ROS, which damage proteins, lipids and DNA but also act as danger signals that activate the innate immune system. Persistent inflammation driven by immune cell infiltration further amplifies oxidative damage through the release of pro‐inflammatory cytokines and additional ROS. Therefore, the vicious cycle of oxidative damage and inflammation contributes significantly to muscle degeneration, fibrosis and impaired regeneration in DMD. Understanding and targeting this molecular crosstalk (Figure 3; Table 1) offers promising therapeutic avenues aimed at preserving muscle function and delaying disease progression in DMD.

Oxidative stress strongly correlates with the severity of the disease [116, 117]. Among the various targets of ROS, disulfide bonds are particularly affected. In 2021, Careccia and colleagues identified high‐mobility group box 1 (HMGB1), one of the most studied damage‐associated molecular patterns (DAMPs), as a critical redox‐sensitive mediator in MDs. The balance between HMGB1 redox isoforms was found to be switched toward the disulfide‐HMGB1, an oxidised isoform, in both muscles isolated from mice models of MDs as well as in patients' biopsies. Moreover, the disulfide form of HMGB1 (dsHMGB1) was found to exacerbate the dystrophic phenotype by increasing necrosis, inflammation and impairing muscle functionality. As a therapeutic approach, they rebalanced HMGB1 redox isoforms toward the regenerative form to counteract the dystrophic phenotype [118]. In particular, *mdx* mice and *Sgca−/−* mice were treated with 3S‐HMGB1, an engineered non‐oxidisable variant of HMGB1, proved to accelerate regeneration after acute damage by acting on different cell types [119]. 3S administration in dystrophic mice models stimulated skeletal muscle regeneration by increasing the percentage of CNM while decreasing degenerating myofibres, mitigating the dystrophic phenotype by dampening inflammation, fibrosis and improving scores of functional tests [118].

Other antioxidant approaches primarily act by slowing degeneration and regeneration cycles. Aminoguanidine hemisulfate [120], an inhibitor of nitric oxide synthase and ROS production, reduced the number of CNM in *mdx* mice, indicating decreased degeneration/regeneration cycles. Similarly, nutraceuticals, with anti‐inflammatory and anti‐oxidant properties, such as Cyanidin 3‐glucoside [121] or polyphenol mixtures like ProAbe [122], attenuated inflammation, fibrosis and necrosis while promoting oxidative metabolism. Notably, cyanidin treatment decreased the number of CNM and reduced calcifications, supporting its role in delaying cycles of degeneration and regeneration [57]. Plumbagin [123], a plant‐derived analogue of vitamin K3 and Benfotiamine [124], a lipid soluble Vitamin B1 analogue, further demonstrated functional and histological benefits by reducing oxidative damage and necrosis, differently affecting skeletal muscle regeneration. Last, epigenetic modulation using JQ1 [125], a BRD4 inhibitor, normalised NADPH oxidase levels, decreased muscle degeneration, reduced ROS and necrosis, restored autophagy and improved physical performance. Most of these approaches highlight the capacity of antioxidant strategies to ameliorate the dystrophic phenotype by dampening oxidative stress and slowing cycles of degeneration and regeneration.

In summary, therapeutic strategies targeting oxidative stress and inflammation in DMD converge on the regulation of degeneration and regeneration dynamics. Antioxidants and metabolic modulators, such as aminoguanidine, cyanidin and BRD4 inhibitors, inhibit ROS production, thereby decreasing the number of CNM and fibrosis. By reducing the frequency of degeneration–regeneration cycles, these interventions may favour the maintenance of SCs in a more quiescent state, thereby preserving the stem cell pool and preventing premature exhaustion. In this context, reduced regeneration does not necessarily reflect impaired repair, but rather a decreased need for compensatory myogenesis due to improved muscle stability. Conversely, approaches like 3S‐HMGB1 increase CNM and enhance CSA, stimulating regeneration and pushing the repair process while mitigating necrosis and inflammation. Importantly, the concomitant reduction in necrosis and inflammation suggests that SC engagement occurs in a more permissive environment, potentially allowing efficient regeneration without triggering chronic overstimulation. Together, these findings underscore that both delaying degeneration and promoting regeneration are complementary strategies, and their balanced integration may offer the most effective route to slow DMD progression and preserve muscle function.

## 7 Conclusions and Perspectives

DMD remains an incurable disease, with a relentlessly progressive and ultimately fatal course. Although gene‐based strategies target the primary genetic defect and cell‐based approaches aim to restore dystrophin expression and muscle homeostasis, substantial challenges remain before their full clinical potential can be realised.

Therefore, several therapeutic strategies have focused on preserving muscle quality, both morphologically and functionally, by limiting tissue damage and supporting residual regenerative capacity.

In DMD, the continuous cycle of myofibre degeneration and incomplete regeneration is a hallmark of disease progression. This chronic tissue turnover results in progressive muscle wasting and loss of strength. Historically, therapeutic efforts focused on stimulating regeneration. However, growing evidence demonstrates that most of the approaches proposed so far aim instead to slow down the cycles of degeneration and regeneration, targeting various cell types, primarily myofibres and immune cells.

One prominent example is Vamorolone [113], a steroidal anti‐inflammatory compound that reduces inflammation while avoiding many of the side effects associated with the standard care glucocorticoids. While it does not appear to activate directly muscle regeneration, its ability to dampen chronic inflammation creates a muscle microenvironment that is more permissive to regeneration rather than fibrotic repair.

Other pharmacological agents, such as Resolvin‐D2 [99] and 3S‐HMGB1 [118], are notable for their dual anti‐inflammatory and pro‐regenerative roles. These compounds not only accelerate the resolution phase of inflammation but also promote the formation of new myofibres, facilitating effective regeneration. Conversely, alternative strategies, such as the inhibition of transcription factor NFIX [55, 102], act by delaying macrophages' skewing toward an anti‐inflammatory phenotype and simultaneously by slowing myogenesis.

Interestingly, the punctual modulation of different processes occurring within skeletal muscle was revealed to be beneficial in preclinical settings. Therapies to stabilise myofibres, mitochondrial well‐being and most of the anti‐oxidant and anti‐inflammatory are associated with a reduction in the number of CNM and fibrofatty deposition, demonstrating a delay in the degeneration cycles, preserving muscle integrity and reflecting a decreased need for compensatory regeneration. On the contrary, an increase of CNM and enhancement of CSA lead to better muscle morphology and functionality. Therefore, stimulating regeneration can rapidly repair muscle tissue, but it must be balanced with the protection of degenerative cycles and the SC pool exhaustion. This highlights the importance not just of modulating the overall cycle but also of synchronising the timing of key processes in a coordinated manner. Together, these observations highlight that therapeutic efficacy in DMD depends not only on modulating the extent of regeneration or degeneration but also critically on coordinating their timing, allowing regeneration to proceed when tissue damage is controlled and the stem cell niche is preserved.

While these strategies do not restore dystrophin expression, they play a pivotal role in maintaining muscle integrity and functionality, thereby improving patients' quality of life. Importantly, by mitigating inflammation, fibrosis and excessive degeneration, they help preserve a permissive muscle environment. This creates a crucial therapeutic window in which more definitive interventions, such as AAV‐mediated micro dystrophin delivery, CRISPR‐based exon skipping or stem cell transplantation, can be applied with higher chances of success, as they rely on a niche that is more conducive to regeneration than to scarring.

To conclude, while dystrophin restoration will undoubtedly remain the cornerstone of disease‐modifying therapies in DMD, it is increasingly clear that no single intervention can fully address the multifaceted nature of the disease. The field now stands at a decisive juncture: efforts to curb degeneration, preserve the regenerative stem cell niche and stabilise the sarcolemma must be woven together into integrated, synergistic strategies. The true transformative potential lies in combining approaches that accelerate SC differentiation and sustain their long‐term fitness with complementary interventions that reinforce myofibre stability, preserve calcium homeostasis, and rebalance oxidative stress and inflammation. Such multidimensional interventions hold the promise not only of reducing toxicity and enhancing efficacy but also of reshaping the therapeutic landscape of DMD.

We call upon the research community to embrace this paradigm of synergy, uniting gene‐ and cell‐based restoration of dystrophin with complementary, pathway‐specific interventions. This integrated vision represents the most promising horizon for the next generation of preclinical and clinical efforts, and the path most likely to deliver durable, comprehensive and life‐changing benefits to patients.

## Conflicts of Interest

The authors declare no conflicts of interest.

## References

[1] E. Mercuri , C. G. Bonnemann , and F. Muntoni , “Muscular Dystrophies,” Lancet 394, no. 10213 (2019): 2025–2038. 31789220 10.1016/S0140-6736(19)32910-1

[2] M. Chang , Y. Cai , Z. Gao , et al., “Duchenne Muscular Dystrophy: Pathogenesis and Promising Therapies,” Journal of Neurology 270, no. 8 (2023): 3733–3749. 37258941 10.1007/s00415-023-11796-x

[3] T. Hoang and R. A. E. Dowdy , “A Review of Muscular Dystrophies,” Anesthesia Progress 71, no. 1 (2024): 44–52. 39503119 10.2344/673191 PMC11101287

[4] A. Bez Batti Angulski , N. Hosny , H. Cohen , et al., “Duchenne Muscular Dystrophy: Disease Mechanism and Therapeutic Strategies,” Frontiers in Physiology 14 (2023): 1183101. 37435300 10.3389/fphys.2023.1183101 PMC10330733

[5] M. D. Grounds , J. R. Terrill , B. A. al‐Mshhdani , M. N. Duong , H. G. Radley‐Crabb , and P. G. Arthur , “Biomarkers for Duchenne Muscular Dystrophy: Myonecrosis, Inflammation and Oxidative Stress,” Disease Models & Mechanisms 13, no. 2 (2020): dmm043638. 32224496 10.1242/dmm.043638 PMC7063669

[6] G. Careccia , L. Mangiavini , and F. Cirillo , “Regulation of Satellite Cells Functions During Skeletal Muscle Regeneration: A Critical Step in Physiological and Pathological Conditions,” International Journal of Molecular Sciences 25, no. 1 (2023): 512. 38203683 10.3390/ijms25010512 PMC10778731

[7] L. Krishna , A. Prashant , Y. H. Kumar , et al., “Molecular and Biochemical Therapeutic Strategies for Duchenne Muscular Dystrophy,” Neurology International 16, no. 4 (2024): 731–760. 39051216 10.3390/neurolint16040055 PMC11270304

[8] T. Tominari , C. Sathyaprakash , and Y. Aoki , “Stem/Progenitor Cell‐Based Therapy for Duchenne Muscular Dystrophy,” Frontiers in Cell and Development Biology 13 (2025): 1640275. 10.3389/fcell.2025.1640275 PMC12441038 40970088

[9] N. Elangkovan and G. Dickson , “Gene Therapy for Duchenne Muscular Dystrophy,” Journal of Neuromuscular Diseases 8, no. s2 (2021): S303–S316. 34511510 10.3233/JND-210678 PMC8673537

[10] D. G. Allen , et al., “Calcium and the Damage Pathways in Muscular Dystrophy,” Canadian Journal of Physiology and Pharmacology 88, no. 2 (2010): 83–91. 10.1139/Y09-058 20237582

[11] A. E. Rossi and R. T. Dirksen , “Sarcoplasmic Reticulum: The Dynamic Calcium Governor of Muscle,” Muscle & Nerve 33, no. 6 (2006): 715–731. 16477617 10.1002/mus.20512

[12] S. A. Goonasekera , C. K. Lam , D. P. Millay , et al., “Mitigation of Muscular Dystrophy in Mice by SERCA Overexpression in Skeletal Muscle,” Journal of Clinical Investigation 121, no. 3 (2011): 1044–1052. 21285509 10.1172/JCI43844 PMC3049367

[13] K. Nogami , Y. Maruyama , F. Sakai‐Takemura , et al., “Pharmacological Activation of SERCA Ameliorates Dystrophic Phenotypes in Dystrophin‐Deficient mdx Mice,” Human Molecular Genetics 30, no. 11 (2021): 1006–1019. 33822956 10.1093/hmg/ddab100 PMC8170845

[14] D. P. Millay , M. A. Sargent , H. Osinska , et al., “Genetic and Pharmacologic Inhibition of Mitochondrial‐Dependent Necrosis Attenuates Muscular Dystrophy,” Nature Medicine 14, no. 4 (2008): 442–447. 10.1038/nm1736 PMC2655270 18345011

[15] E. R. Wissing , D. P. Millay , G. Vuagniaux , and J. D. Molkentin , “Debio‐025 Is More Effective Than Prednisone in Reducing Muscular Pathology in mdx Mice,” Neuromuscular Disorders 20, no. 11 (2010): 753–760. 20637615 10.1016/j.nmd.2010.06.016 PMC2980760

[16] M. V. Dubinin , V. S. Starinets , E. Y. Talanov , I. B. Mikheeva , N. V. Belosludtseva , and K. N. Belosludtsev , “Alisporivir Improves Mitochondrial Function in Skeletal Muscle of mdx Mice but Suppresses Mitochondrial Dynamics and Biogenesis,” International Journal of Molecular Sciences 22, no. 18 (2021): 9780. 34575944 10.3390/ijms22189780 PMC8464657

[17] M. V. Dubinin , A. E. Stepanova , I. B. Mikheeva , et al., “Reduction of Mitochondrial Calcium Overload via MKT077‐Induced Inhibition of Glucose‐Regulated Protein 75 Alleviates Skeletal Muscle Pathology in Dystrophin‐Deficient mdx Mice,” International Journal of Molecular Sciences 25, no. 18 (2024): 9892. 39337383 10.3390/ijms25189892 PMC11432509

[18] M. V. Dubinin , V. S. Starinets , N. V. Belosludtseva , et al., “BK (Ca) Activator NS1619 Improves the Structure and Function of Skeletal Muscle Mitochondria in Duchenne Dystrophy,” Pharmaceutics 14, no. 11 (2022): 2336. 36365155 10.3390/pharmaceutics14112336 PMC9696041

[19] J. Lin , C. Handschin , and B. M. Spiegelman , “Metabolic Control Through the PGC‐1 Family of Transcription Coactivators,” Cell Metabolism 1, no. 6 (2005): 361–370. 16054085 10.1016/j.cmet.2005.05.004

[20] C. Handschin , Y. M. Kobayashi , S. Chin , P. Seale , K. P. Campbell , and B. M. Spiegelman , “PGC‐1alpha Regulates the Neuromuscular Junction Program and Ameliorates Duchenne Muscular Dystrophy,” Genes & Development 21, no. 7 (2007): 770–783. 17403779 10.1101/gad.1525107 PMC1838529

[21] C. Webster , L. Silberstein , A. P. Hays , and H. M. Blau , “Fast Muscle Fibers Are Preferentially Affected in Duchenne Muscular Dystrophy,” Cell 52, no. 4 (1988): 503–513. 3342447 10.1016/0092-8674(88)90463-1

[22] M. C. Chan , G. C. Rowe , S. Raghuram , I. S. Patten , C. Farrell , and Z. Arany , “Post‐Natal Induction of PGC‐1alpha Protects Against Severe Muscle Dystrophy Independently of Utrophin,” Skeletal Muscle 4, no. 1 (2014): 2. 24447845 10.1186/2044-5040-4-2 PMC3914847

[23] J. Gonzalez‐Sanchez , A. Sánchez‐Temprano , T. Cid‐Díaz , et al., “Improvement of Duchenne Muscular Dystrophy Phenotype Following Obestatin Treatment,” Journal of Cachexia, Sarcopenia and Muscle 9, no. 6 (2018): 1063–1078. 30216693 10.1002/jcsm.12338 PMC6240759

[24] C. Canto and J. Auwerx , “PGC‐1alpha, SIRT1 and AMPK, an Energy Sensing Network That Controls Energy Expenditure,” Current Opinion in Lipidology 20, no. 2 (2009): 98–105. 19276888 10.1097/MOL.0b013e328328d0a4 PMC3627054

[25] R. Mounier , M. Théret , L. Lantier , M. Foretz , and B. Viollet , “Expanding Roles for AMPK in Skeletal Muscle Plasticity,” Trends in Endocrinology and Metabolism 26, no. 6 (2015): 275–286. 25818360 10.1016/j.tem.2015.02.009

[26] L. Barre , C. Richardson , M. F. Hirshman , et al., “Genetic Model for the Chronic Activation of Skeletal Muscle AMP‐Activated Protein Kinase Leads to Glycogen Accumulation,” American Journal of Physiology. Endocrinology and Metabolism 292, no. 3 (2007): E802–E811. 17106064 10.1152/ajpendo.00369.2006

[27] V. Ljubicic , P. Miura , M. Burt , et al., “Chronic AMPK Activation Evokes the Slow, Oxidative Myogenic Program and Triggers Beneficial Adaptations in mdx Mouse Skeletal Muscle,” Human Molecular Genetics 20, no. 17 (2011): 3478–3493. 21659335 10.1093/hmg/ddr265

[28] S. Y. Ng , A. I. Mikhail , S. R. Mattina , A. Manta , I. J. Diffey , and V. Ljubicic , “Acute, Next‐Generation AMPK Activation Initiates a Disease‐Resistant Gene Expression Program in Dystrophic Skeletal Muscle,” FASEB Journal 37, no. 5 (2023): e22863. 37016990 10.1096/fj.202201846RR

[29] A. Chalkiadaki , M. Igarashi , A. S. Nasamu , J. Knezevic , and L. Guarente , “Muscle‐Specific SIRT1 Gain‐of‐Function Increases Slow‐Twitch Fibers and Ameliorates Pathophysiology in a Mouse Model of Duchenne Muscular Dystrophy,” PLoS Genetics 10, no. 7 (2014): e1004490. 25032964 10.1371/journal.pgen.1004490 PMC4102452

[30] M. Giovarelli , S. Zecchini , S. R. Casati , et al., “The SIRT1 Activator SRT2104 Exerts Exercise Mimetic Effects and Promotes Duchenne Muscular Dystrophy Recovery,” Cell Death & Disease 16, no. 1 (2025): 259. 40195304 10.1038/s41419-025-07595-z PMC11977210

[31] V. Ljubicic , M. Burt , J. A. Lunde , and B. J. Jasmin , “Resveratrol Induces Expression of the Slow, Oxidative Phenotype in mdx Mouse Muscle Together With Enhanced Activity of the SIRT1‐PGC‐1alpha Axis,” American Journal of Physiology. Cell Physiology 307, no. 1 (2014): C66–C82. 24760981 10.1152/ajpcell.00357.2013 PMC4080183

[32] R. Sebori , A. Kuno , R. Hosoda , T. Hayashi , and Y. Horio , “Resveratrol Decreases Oxidative Stress by Restoring Mitophagy and Improves the Pathophysiology of Dystrophin‐Deficient mdx Mice,” Oxidative Medicine and Cellular Longevity 2018 (2018): 9179270. 30510631 10.1155/2018/9179270 PMC6231358

[33] K. Kawamura , S. Fukumura , K. Nikaido , et al., “Resveratrol Improves Motor Function in Patients With Muscular Dystrophies: an Open‐Label, Single‐Arm, Phase IIa Study,” Scientific Reports 10, no. 1 (2020): 20585. 33239684 10.1038/s41598-020-77197-6 PMC7688653

[34] P. Miura , J. V. Chakkalakal , L. Boudreault , et al., “Pharmacological Activation of PPARbeta/Delta Stimulates Utrophin A Expression in Skeletal Muscle Fibers and Restores Sarcolemmal Integrity in Mature mdx Mice,” Human Molecular Genetics 18, no. 23 (2009): 4640–4649. 19744959 10.1093/hmg/ddp431

[35] V. E. Jahnke , J. H. van der Meulen , H. K. Johnston , et al., “Metabolic Remodeling Agents Show Beneficial Effects in the Dystrophin‐Deficient mdx Mouse Model,” Skeletal Muscle 2, no. 1 (2012): 16. 22908954 10.1186/2044-5040-2-16 PMC3482394

[36] B. Lagu , A. F. Kluge , E. Tozzo , et al., “Selective PPARdelta Modulators Improve Mitochondrial Function: Potential Treatment for Duchenne Muscular Dystrophy (DMD),” ACS Medicinal Chemistry Letters 9, no. 9 (2018): 935–940. 30258544 10.1021/acsmedchemlett.8b00287 PMC6142063

[37] M. Ito , S. Tauscher‐Wisniewski , R. A. Smulders , et al., “Single‐ and Multiple‐Dose Safety, Tolerability, Pharmacokinetic, and Pharmacodynamic Profiles of ASP0367, or Bocidelpar Sulfate, a Novel Modulator of Peroxisome Proliferator‐Activated Receptor Delta in Healthy Adults: Results From a Phase 1 Study,” Muscle & Nerve 65, no. 1 (2022): 110–120. 34642949 10.1002/mus.27436 PMC9298414

[38] K. Hnia , J. Gayraud , G. Hugon , et al., “L‐arginine Decreases Inflammation and Modulates the Nuclear Factor‐KappaB/Matrix Metalloproteinase Cascade in mdx Muscle Fibers,” American Journal of Pathology 172, no. 6 (2008): 1509–1519. 18458097 10.2353/ajpath.2008.071009 PMC2408412

[39] G. Juban , M. Saclier , H. Yacoub‐Youssef , et al., “AMPK Activation Regulates LTBP4‐Dependent TGF‐beta1 Secretion by Pro‐inflammatory Macrophages and Controls Fibrosis in Duchenne Muscular Dystrophy,” Cell Reports 25, no. 8 (2018): 2163–2176 e6. 30463013 10.1016/j.celrep.2018.10.077

[40] P. Hafner , U. Bonati , D. Rubino , et al., “Treatment With L‐Citrulline and Metformin in Duchenne Muscular Dystrophy: Study Protocol for a Single‐Centre, Randomised, Placebo‐Controlled Trial,” Trials 17, no. 1 (2016): 389. 27488051 10.1186/s13063-016-1503-1 PMC4973063

[41] P. Hafner , U. Bonati , A. Klein , et al., “Effect of Combination l‐Citrulline and Metformin Treatment on Motor Function in Patients With Duchenne Muscular Dystrophy: A Randomized Clinical Trial,” JAMA Network Open 2, no. 10 (2019): e1914171. 31664444 10.1001/jamanetworkopen.2019.14171 PMC6824222

[42] P. Luan , D. D’Amico , P. A. Andreux , et al., “Urolithin A Improves Muscle Function by Inducing Mitophagy in Muscular Dystrophy,” Science Translational Medicine 13, no. 588 (2021): eabb0319. 33827972 10.1126/scitranslmed.abb0319

[43] J. Tinsley , N. Deconinck , R. Fisher , et al., “Expression of Full‐Length Utrophin Prevents Muscular Dystrophy in mdx Mice,” Nature Medicine 4, no. 12 (1998): 1441–1444. 10.1038/4033 9846586

[44] J. M. Tinsley , R. J. Fairclough , R. Storer , et al., “Daily Treatment With SMTC1100, a Novel Small Molecule Utrophin Upregulator, Dramatically Reduces the Dystrophic Symptoms in the mdx Mouse,” PLoS ONE 6, no. 5 (2011): e19189. 21573153 10.1371/journal.pone.0019189 PMC3089598

[45] M. Chatzopoulou , T. D. W. Claridge , K. E. Davies , et al., “Isolation, Structural Identification, Synthesis, and Pharmacological Profiling of 1,2‐Trans‐Dihydro‐1,2‐diol Metabolites of the Utrophin Modulator Ezutromid,” Journal of Medicinal Chemistry 63, no. 5 (2020): 2547–2556. 31599580 10.1021/acs.jmedchem.9b01547

[46] R. Wu , P. Li , P. Xiao , et al., “Activation of Endogenous Full‐Length Utrophin by MyoAAV‐UA as a Therapeutic Approach for Duchenne Muscular Dystrophy,” Nature Communications 16, no. 1 (2025): 2398. 10.1038/s41467-025-57831-5 PMC11894210 40064877

[47] A. J. Russell , M. DuVall , B. Barthel , et al., “Modulating Fast Skeletal Muscle Contraction Protects Skeletal Muscle in Animal Models of Duchenne Muscular Dystrophy,” Journal of Clinical Investigation 133, no. 10 (2023): e153837. 36995778 10.1172/JCI153837 PMC10178848

[48] J. Donovan , J. A. Silverman , B. Barthel , et al., “A Phase 1, Double‐Blind, Placebo‐Controlled Trial of Sevasemten (EDG‐5506), a Selective Modulator of Fast Skeletal Muscle Contraction, in Healthy Volunteers and Adults With Becker Muscular Dystrophy,” Muscle & Nerve 72, no. 3 (2025): 399–407. 40452637 10.1002/mus.28444 PMC12338012

[49] A. Bonato , G. Raparelli , S. Luvisetto , et al., “Cyclin D3 Deficiency Promotes a Slower, More Oxidative Skeletal Muscle Phenotype and Ameliorates Pathophysiology in the mdx Mouse Model of Duchenne Muscular Dystrophy,” FASEB Journal 37, no. 7 (2023): e23025. 37309599 10.1096/fj.202201769R

[50] N. L. Reyes , G. B. Banks , M. Tsang , et al., “Fnip1 Regulates Skeletal Muscle Fiber Type Specification, Fatigue Resistance, and Susceptibility to Muscular Dystrophy,” Proceedings of the National Academy of Sciences of the United States of America 112, no. 2 (2015): 424–429. 25548157 10.1073/pnas.1413021112 PMC4299192

[51] J. E. Darnell, Jr. , “Transcription Factors as Targets for Cancer Therapy,” Nature Reviews. Cancer 2, no. 10 (2002): 740–749. 12360277 10.1038/nrc906

[52] A. Chen and A. N. Koehler , “Transcription Factor Inhibition: Lessons Learned and Emerging Targets,” Trends in Molecular Medicine 26, no. 5 (2020): 508–518. 32359481 10.1016/j.molmed.2020.01.004 PMC7198608

[53] E. Blanchet , J. S. Annicotte , S. Lagarrigue , et al., “E2F Transcription Factor‐1 Regulates Oxidative Metabolism,” Nature Cell Biology 13, no. 9 (2011): 1146–1152. 21841792 10.1038/ncb2309 PMC3849758

[54] E. Blanchet , J. S. Annicotte , L. A. Pradelli , et al., “E2F Transcription Factor‐1 Deficiency Reduces Pathophysiology in the Mouse Model of Duchenne Muscular Dystrophy Through Increased Muscle Oxidative Metabolism,” Human Molecular Genetics 21, no. 17 (2012): 3910–3917. 22678059 10.1093/hmg/dds219 PMC3412384

[55] G. Rossi , C. Bonfanti , S. Antonini , et al., “Silencing Nfix Rescues Muscular Dystrophy by Delaying Muscle Regeneration,” Nature Communications 8, no. 1 (2017): 1055. 10.1038/s41467-017-01098-y PMC5651883 29057908

[56] V. Taglietti , G. Angelini , G. Mura , et al., “RhoA and ERK Signalling Regulate the Expression of the Transcription Factor Nfix in Myogenic Cells,” Development 145, no. 21 (2018): dev163956. 30266829 10.1242/dev.163956

[57] G. Angelini , E. Capra , F. Rossi , et al., “MEK‐Inhibitors Decrease Nfix in Muscular Dystrophy but Induce Unexpected Calcifications, Partially Rescued With Cyanidin Diet,” iScience 27, no. 1 (2024): 108696. 38205246 10.1016/j.isci.2023.108696 PMC10777118

[58] M. Yoshida , S. Horinouchi , and T. Beppu , “Trichostatin A and Trapoxin: Novel Chemical Probes for the Role of Histone Acetylation in Chromatin Structure and Function,” BioEssays 17, no. 5 (1995): 423–430. 7786288 10.1002/bies.950170510

[59] C. Colussi , C. Mozzetta , A. Gurtner , et al., “HDAC2 Blockade by Nitric Oxide and Histone Deacetylase Inhibitors Reveals a Common Target in Duchenne Muscular Dystrophy Treatment,” Proceedings of the National Academy of Sciences of the United States of America 105, no. 49 (2008): 19183–19187. 19047631 10.1073/pnas.0805514105 PMC2614736

[60] J. Vojinovic , N. Damjanov , C. D'Urzo , et al., “Safety and Efficacy of an Oral Histone Deacetylase Inhibitor in Systemic‐Onset Juvenile Idiopathic Arthritis,” Arthritis and Rheumatism 63, no. 5 (2011): 1452–1458. 21538322 10.1002/art.30238

[61] S. Consalvi , C. Mozzetta , P. Bettica , et al., “Preclinical Studies in the mdx Mouse Model of Duchenne Muscular Dystrophy With the Histone Deacetylase Inhibitor Givinostat,” Molecular Medicine 19, no. 1 (2013): 79–87. 23552722 10.2119/molmed.2013.00011 PMC3667212

[62] P. Bettica , S. Petrini , V. D'Oria , et al., “Histological Effects of Givinostat in Boys With Duchenne Muscular Dystrophy,” Neuromuscular Disorders 26, no. 10 (2016): 643–649. 27566866 10.1016/j.nmd.2016.07.002

[63] E. Mercuri , J. J. Vilchez , O. Boespflug‐Tanguy , et al., “Safety and Efficacy of Givinostat in Boys With Duchenne Muscular Dystrophy (EPIDYS): a Multicentre, Randomised, Double‐Blind, Placebo‐Controlled, Phase 3 Trial,” Lancet Neurology 23, no. 4 (2024): 393–403. 38508835 10.1016/S1474-4422(24)00036-X

[64] A. Osseni , A. Ravel‐Chapuis , E. Belotti , et al., “Pharmacological Inhibition of HDAC6 Improves Muscle Phenotypes in Dystrophin‐Deficient Mice by Downregulating TGF‐Beta via Smad3 Acetylation,” Nature Communications 13, no. 1 (2022): 7108. 10.1038/s41467-022-34831-3 PMC9675748 36402791

[65] A. C. McPherron , A. M. Lawler , and S. J. Lee , “Regulation of Skeletal Muscle Mass in Mice by a New TGF‐Beta Superfamily Member,” Nature 387, no. 6628 (1997): 83–90. 9139826 10.1038/387083a0

[66] M. H. Baig , K. Ahmad , J. S. Moon , et al., “Myostatin and Its Regulation: A Comprehensive Review of Myostatin Inhibiting Strategies,” Frontiers in Physiology 13 (2022): 876078. 35812316 10.3389/fphys.2022.876078 PMC9259834

[67] S. Bogdanovich , T. O. B. Krag , E. R. Barton , et al., “Functional Improvement of Dystrophic Muscle by Myostatin Blockade,” Nature 420, no. 6914 (2002): 418–421. 12459784 10.1038/nature01154

[68] A. Iskenderian , N. Liu , Q. Deng , et al., “Myostatin and Activin Blockade by Engineered Follistatin Results in Hypertrophy and Improves Dystrophic Pathology in mdx Mouse More Than Myostatin Blockade Alone,” Skeletal Muscle 8, no. 1 (2018): 34. 30368252 10.1186/s13395-018-0180-z PMC6204036

[69] M. St Andre , M. Johnson , P. N. Bansal , et al., “A Mouse Anti‐Myostatin Antibody Increases Muscle Mass and Improves Muscle Strength and Contractility in the mdx Mouse Model of Duchenne Muscular Dystrophy and Its Humanized Equivalent, Domagrozumab (PF‐06252616), Increases Muscle Volume in Cynomolgus Monkeys,” Skeletal Muscle 7, no. 1 (2017): 25. 29121992 10.1186/s13395-017-0141-y PMC5679155

[70] P. Singh , H. Rong , T. Gordi , J. Bosley , and I. Bhattacharya , “Translational Pharmacokinetic/Pharmacodynamic Analysis of MYO‐029 Antibody for Muscular Dystrophy,” Clinical and Translational Science 9, no. 6 (2016): 302–310. 27700008 10.1111/cts.12420 PMC5351001

[71] K. R. Wagner , J. L. Fleckenstein , A. A. Amato , et al., “A Phase I/II Trial of MYO‐029 in Adult Subjects With Muscular Dystrophy,” Annals of Neurology 63, no. 5 (2008): 561–571. 18335515 10.1002/ana.21338

[72] K. R. Wagner , H. Z. Abdel‐Hamid , J. K. Mah , et al., “Randomized Phase 2 Trial and Open‐Label Extension of Domagrozumab in Duchenne Muscular Dystrophy,” Neuromuscular Disorders 30, no. 6 (2020): 492–502. 32522498 10.1016/j.nmd.2020.05.002

[73] H. Amthor , R. Macharia , R. Navarrete , et al., “Lack of Myostatin Results in Excessive Muscle Growth but Impaired Force Generation,” Proceedings of the National Academy of Sciences of the United States of America 104, no. 6 (2007): 1835–1840. 17267614 10.1073/pnas.0604893104 PMC1794294

[74] A. Musaro , et al., “Localized Igf‐1 Transgene Expression Sustains Hypertrophy and Regeneration in Senescent Skeletal Muscle,” Nature Genetics 27, no. 2 (2001): 195–200. 11175789 10.1038/84839

[75] A. Musaro , C. Giacinti , G. Borsellino , et al., “Stem Cell‐Mediated Muscle Regeneration Is Enhanced by Local Isoform of Insulin‐Like Growth Factor 1,” Proceedings of the National Academy of Sciences of the United States of America 101, no. 5 (2004): 1206–1210. 14745025 10.1073/pnas.0303792101 PMC337031

[76] M. Feron , L. Guevel , K. Rouger , et al., “PTEN Contributes to Profound PI3K/Akt Signaling Pathway Deregulation in Dystrophin‐Deficient Dog Muscle,” American Journal of Pathology 174, no. 4 (2009): 1459–1470. 19264909 10.2353/ajpath.2009.080460 PMC2671376

[77] P. Gregorevic , D. R. Plant , and G. S. Lynch , “Administration of Insulin‐Like Growth Factor‐I Improves Fatigue Resistance of Skeletal Muscles From Dystrophic mdx Mice,” Muscle & Nerve 30, no. 3 (2004): 295–304. 15318340 10.1002/mus.20082

[78] J. D. Schertzer , J. G. Ryall , and G. S. Lynch , “Systemic Administration of IGF‐I Enhances Oxidative Status and Reduces Contraction‐Induced Injury in Skeletal Muscles of mdx Dystrophic Mice,” American Journal of Physiology. Endocrinology and Metabolism 291, no. 3 (2006): E499–E505. 16621899 10.1152/ajpendo.00101.2006

[79] M. S. Alexander , J. C. Casar , N. Motohashi , et al., “MicroRNA‐486‐Dependent Modulation of DOCK3/PTEN/AKT Signaling Pathways Improves Muscular Dystrophy‐Associated Symptoms,” Journal of Clinical Investigation 124, no. 6 (2014): 2651–2667. 24789910 10.1172/JCI73579 PMC4038577

[80] H. J. Kok and E. R. Barton , “Actions and Interactions of IGF‐I and MMPs During Muscle Regeneration,” Seminars in Cell & Developmental Biology 119 (2021): 11–22. 33962867 10.1016/j.semcdb.2021.04.018 PMC9128303

[81] T. Shan , J. Liu , Z. Xu , and Y. Wang , “Roles of Phosphatase and Tensin Homolog in Skeletal Muscle,” Journal of Cellular Physiology 234, no. 4 (2019): 3192–3196. 30471096 10.1002/jcp.26820

[82] F. Yue , P. Bi , C. Wang , et al., “Pten Is Necessary for the Quiescence and Maintenance of Adult Muscle Stem Cells,” Nature Communications 8 (2017): 14328. 10.1038/ncomms14328 PMC5247606 28094257

[83] O. M. Dorchies , J. Reutenauer‐Patte , E. Dahmane , et al., “The Anticancer Drug Tamoxifen Counteracts the Pathology in a Mouse Model of Duchenne Muscular Dystrophy,” American Journal of Pathology 182, no. 2 (2013): 485–504. 23332367 10.1016/j.ajpath.2012.10.018

[84] D. P. Millay , J. R. O’Rourke , L. B. Sutherland , et al., “Myomaker Is a Membrane Activator of Myoblast Fusion and Muscle Formation,” Nature 499, no. 7458 (2013): 301–305. 23868259 10.1038/nature12343 PMC3739301

[85] M. J. Petrany , T. Song , S. Sadayappan , and D. P. Millay , “Myocyte‐Derived Myomaker Expression Is Required for Regenerative Fusion but Exacerbates Membrane Instability in Dystrophic Myofibers,” JCI Insight 5, no. 9 (2020): e136095. 32310830 10.1172/jci.insight.136095 PMC7253022

[86] J. G. Boyer , J. Huo , S. Han , et al., “Depletion of Skeletal Muscle Satellite Cells Attenuates Pathology in Muscular Dystrophy,” Nature Communications 13, no. 1 (2022): 2940. 10.1038/s41467-022-30619-7 PMC9135721 35618700

[87] T. M. Fontelonga , B. Jordan , A. M. Nunes , et al., “Sunitinib Promotes Myogenic Regeneration and Mitigates Disease Progression in the mdx Mouse Model of Duchenne Muscular Dystrophy,” Human Molecular Genetics 28, no. 13 (2019): 2120–2132. 30806670 10.1093/hmg/ddz044 PMC6586148

[88] J. von Maltzahn , J. M. Renaud , G. Parise , and M. A. Rudnicki , “Wnt7a Treatment Ameliorates Muscular Dystrophy,” Proceedings of the National Academy of Sciences of the United States of America 109, no. 50 (2012): 20614–20619. 23185011 10.1073/pnas.1215765109 PMC3528612

[89] L. Matias‐Valiente , C. Sanchez‐Fernandez , L. Rodriguez‐Outeiriño , et al., “Evaluation of Pro‐Regenerative and Anti‐Inflammatory Effects of Isolecanoric Acid in the Muscle: Potential Treatment of Duchenne Muscular Dystrophy,” Biomedicine & Pharmacotherapy 170 (2024): 116056. 38159372 10.1016/j.biopha.2023.116056

[90] V. Taglietti , K. Kefi , L. Rivera , et al., “Thyroid‐Stimulating Hormone Receptor Signaling Restores Skeletal Muscle Stem Cell Regeneration in Rats With Muscular Dystrophy,” Science Translational Medicine 15, no. 685 (2023): eadd5275. 36857434 10.1126/scitranslmed.add5275

[91] A. I. Cojocaru , K. Kefi , J. D. Masson , L. Tiret , F. Relaix , and V. Taglietti , “Forskolin Treatment Enhances Muscle Regeneration and Shows Therapeutic Potential With Limitations in Duchenne Muscular Dystrophy,” Skeletal Muscle 15, no. 1 (2025): 12. 40329365 10.1186/s13395-025-00381-7 PMC12057055

[92] S. Matsuda and S. Koyasu , “Mechanisms of Action of Cyclosporine,” Immunopharmacology 47, no. 2–3 (2000): 119–125. 10878286 10.1016/s0162-3109(00)00192-2

[93] A. De Luca , B. Nico , A. Liantonio , et al., “A Multidisciplinary Evaluation of the Effectiveness of Cyclosporine a in Dystrophic mdx Mice,” American Journal of Pathology 166, no. 2 (2005): 477–489. 15681831 10.1016/S0002-9440(10)62270-5 PMC1602333

[94] J. Kirschner , J. Schessl , U. Schara , et al., “Treatment of Duchenne Muscular Dystrophy With Ciclosporin A: A Randomised, Double‐Blind, Placebo‐Controlled Multicentre Trial,” Lancet Neurology 9, no. 11 (2010): 1053–1059. 20801085 10.1016/S1474-4422(10)70196-4

[95] J. V. Fernandez‐Montero , E. Vispo , and V. Soriano , “Emerging Antiretroviral Drugs,” Expert Opinion on Pharmacotherapy 15, no. 2 (2014): 211–219. 24289800 10.1517/14656566.2014.863277

[96] S. L. Friedman , V. Ratziu , S. A. Harrison , et al., “A Randomized, Placebo‐Controlled Trial of Cenicriviroc for Treatment of Nonalcoholic Steatohepatitis With Fibrosis,” Hepatology 67, no. 5 (2018): 1754–1767. 28833331 10.1002/hep.29477 PMC5947654

[97] F. Liang , C. Giordano , D. Shang , Q. Li , and B. J. Petrof , “The Dual CCR2/CCR5 Chemokine Receptor Antagonist Cenicriviroc Reduces Macrophage Infiltration and Disease Severity in Duchenne Muscular Dystrophy (Dmdmdx‐4Cv) Mice,” PLoS ONE 13, no. 3 (2018): e0194421. 29561896 10.1371/journal.pone.0194421 PMC5862483

[98] N. Dubuisson , M. A. Davis‐López de Carrizosa , R. Versele , et al., “Inhibiting the Inflammasome With MCC950 Counteracts Muscle Pyroptosis and Improves Duchenne Muscular Dystrophy,” Frontiers in Immunology 13 (2022): 1049076. 36569900 10.3389/fimmu.2022.1049076 PMC9770793

[99] J. Dort , Z. Orfi , P. Fabre , et al., “Resolvin‐D2 Targets Myogenic Cells and Improves Muscle Regeneration in Duchenne Muscular Dystrophy,” Nature Communications 12, no. 1 (2021): 6264. 10.1038/s41467-021-26516-0 PMC8556273 34716330

[100] J. Dort , Z. Orfi , M. Fiscaletti , P. M. Campeau , and N. A. Dumont , “Gpr18 Agonist Dampens Inflammation, Enhances Myogenesis, and Restores Muscle Function in Models of Duchenne Muscular Dystrophy,” Frontiers in Cell and Development Biology 11 (2023): 1187253. 10.3389/fcell.2023.1187253 PMC10461444 37645248

[101] M. Saclier , M. Lapi , C. Bonfanti , G. Rossi , S. Antonini , and G. Messina , “The Transcription Factor Nfix Requires RhoA‐ROCK1 Dependent Phagocytosis to Mediate Macrophage Skewing During Skeletal Muscle Regeneration,” Cells 9, no. 3 (2020): 708. 32183151 10.3390/cells9030708 PMC7140652

[102] M. Saclier , G. Angelini , C. Bonfanti , G. Mura , G. Temponi , and G. Messina , “Selective Ablation of Nfix in Macrophages Attenuates Muscular Dystrophy by Inhibiting Fibro‐Adipogenic Progenitor‐Dependent Fibrosis,” Journal of Pathology 257, no. 3 (2022): 352–366. 35297529 10.1002/path.5895 PMC9322546

[103] W. J. Durham , S. Arbogast , E. Gerken , Y. P. Li , and M. B. Reid , “Progressive Nuclear Factor‐kappaB Activation Resistant to Inhibition by Contraction and Curcumin in mdx Mice,” Muscle & Nerve 34, no. 3 (2006): 298–303. 16718687 10.1002/mus.20579

[104] S. Acharyya , S. A. Villalta , N. Bakkar , et al., “Interplay of IKK/NF‐kappaB Signaling in Macrophages and Myofibers Promotes Muscle Degeneration in Duchenne Muscular Dystrophy,” Journal of Clinical Investigation 117, no. 4 (2007): 889–901. 17380205 10.1172/JCI30556 PMC1821069

[105] R. I. Scheinman , P. C. Cogswell , A. K. Lofquist , and A. S. Baldwin, Jr. , “Role of Transcriptional Activation of I Kappa B Alpha in Mediation of Immunosuppression by Glucocorticoids,” Science 270, no. 5234 (1995): 283–286. 7569975 10.1126/science.270.5234.283

[106] R. Shenkar , H. K. Yum , J. Arcaroli , J. Kupfner , and E. Abraham , “Interactions Between CBP, NF‐kappaB, and CREB in the Lungs After Hemorrhage and Endotoxemia,” American Journal of Physiology. Lung Cellular and Molecular Physiology 281, no. 2 (2001): L418–L426. 11435217 10.1152/ajplung.2001.281.2.L418

[107] E. Solito , A. Mulla , J. F. Morris , H. C. Christian , R. J. Flower , and J. C. Buckingham , “Dexamethasone Induces Rapid Serine‐Phosphorylation and Membrane Translocation of Annexin 1 in a Human Folliculostellate Cell Line via a Novel Nongenomic Mechanism Involving the Glucocorticoid Receptor, Protein Kinase C, Phosphatidylinositol 3‐Kinase, and Mitogen‐Activated Protein Kinase,” Endocrinology 144, no. 4 (2003): 1164–1174. 12639897 10.1210/en.2002-220592

[108] D. B. Drachman , K. V. Toyka , and E. Myer , “Prednisone in Duchenne Muscular Dystrophy,” Lancet 2, no. 7894 (1974): 1409–1412. 4140328 10.1016/s0140-6736(74)90071-3

[109] G. M. Fenichel , J. M. Florence , A. Pestronk , et al., “Long‐Term Benefit From Prednisone Therapy in Duchenne Muscular Dystrophy,” Neurology 41, no. 12 (1991): 1874–1877. 1745340 10.1212/wnl.41.12.1874

[110] A. Sali , A. D. Guerron , H. Gordish‐Dressman , et al., “Glucocorticoid‐Treated Mice Are an Inappropriate Positive Control for Long‐Term Preclinical Studies in the mdx Mouse,” PLoS ONE 7, no. 4 (2012): e34204. 22509280 10.1371/journal.pone.0034204 PMC3317932

[111] M. Wehling‐Henricks , J. J. Lee , and J. G. Tidball , “Prednisolone Decreases Cellular Adhesion Molecules Required for Inflammatory Cell Infiltration in Dystrophin‐Deficient Skeletal Muscle,” Neuromuscular Disorders 14, no. 8–9 (2004): 483–490. 15336689 10.1016/j.nmd.2004.04.008

[112] K. Ma , C. Mallidis , S. Bhasin , et al., “Glucocorticoid‐Induced Skeletal Muscle Atrophy Is Associated With Upregulation of Myostatin Gene Expression,” American Journal of Physiology. Endocrinology and Metabolism 285, no. 2 (2003): E363–E371. 12721153 10.1152/ajpendo.00487.2002

[113] C. R. Heier , J. M. Damsker , Q. Yu , et al., “VBP15, a Novel Anti‐Inflammatory and Membrane‐Stabilizer, Improves Muscular Dystrophy Without Side Effects,” EMBO Molecular Medicine 5, no. 10 (2013): 1569–1585. 24014378 10.1002/emmm.201302621 PMC3799580

[114] L. S. Conklin , J. M. Damsker , E. P. Hoffman , et al., “Phase IIa Trial in Duchenne Muscular Dystrophy Shows Vamorolone Is a First‐in‐Class Dissociative Steroidal Anti‐Inflammatory Drug,” Pharmacological Research 136 (2018): 140–150. 30219580 10.1016/j.phrs.2018.09.007 PMC6218284

[115] R. S. Finkel , C. M. McDonald , H. Lee Sweeney , et al., “A Randomized, Double‐Blind, Placebo‐Controlled, Global Phase 3 Study of Edasalonexent in Pediatric Patients With Duchenne Muscular Dystrophy: Results of the PolarisDMD Trial,” Journal of Neuromuscular Diseases 8, no. 5 (2021): 769–784. 34120912 10.3233/JND-210689 PMC8543277

[116] R. Renjini , N. Gayathri , A. Nalini , and M. M. Srinivas Bharath , “Oxidative Damage in Muscular Dystrophy Correlates With the Severity of the Pathology: Role of Glutathione Metabolism,” Neurochemical Research 37, no. 4 (2012): 885–898. 22219131 10.1007/s11064-011-0683-z

[117] J. R. Terrill , H. G. Radley‐Crabb , T. Iwasaki , F. A. Lemckert , P. G. Arthur , and M. D. Grounds , “Oxidative Stress and Pathology in Muscular Dystrophies: Focus on Protein Thiol Oxidation and Dysferlinopathies,” FEBS Journal 280, no. 17 (2013): 4149–4164. 23332128 10.1111/febs.12142

[118] G. Careccia , M. Saclier , M. Tirone , et al., “Rebalancing Expression of HMGB1 Redox Isoforms to Counteract Muscular Dystrophy,” Science Translational Medicine 13, no. 596 (2021): eaay8416. 34078746 10.1126/scitranslmed.aay8416

[119] M. Tirone , N. L. Tran , C. Ceriotti , et al., “High Mobility Group Box 1 Orchestrates Tissue Regeneration via CXCR4,” Journal of Experimental Medicine 215, no. 1 (2018): 303–318. 29203538 10.1084/jem.20160217 PMC5748844

[120] S. Sun , T. Yu , J. Y. Huh , Y. Cai , S. Yoon , and H. M. A. Javaid , “Aminoguanidine Hemisulfate Improves Mitochondrial Autophagy, Oxidative Stress, and Muscle Force in Duchenne Muscular Dystrophy via the AKT/FOXO1 Pathway in mdx Mice,” Skeletal Muscle 15, no. 1 (2025): 2. 39806512 10.1186/s13395-024-00371-1 PMC11726948

[121] M. Saclier , C. Bonfanti , S. Antonini , et al., “Nutritional Intervention With Cyanidin Hinders the Progression of Muscular Dystrophy,” Cell Death & Disease 11, no. 2 (2020): 127. 32071288 10.1038/s41419-020-2332-4 PMC7028923

[122] C. Sitzia , A. Farini , F. Colleoni , et al., “Improvement of Endurance of DMD Animal Model Using Natural Polyphenols,” BioMed Research International 2015 (2015): 680615. 25861640 10.1155/2015/680615 PMC4377377

[123] D. Cervia , S. Zecchini , L. Pincigher , et al., “Oral Administration of Plumbagin Is Beneficial in In Vivo Models of Duchenne Muscular Dystrophy Through Control of Redox Signaling,” Free Radical Biology & Medicine 225 (2024): 193–207. 39326684 10.1016/j.freeradbiomed.2024.09.037

[124] C. A. Coles , K. G. Woodman , E. M. Gibbs , R. H. Crosbie , J. D. White , and S. R. Lamandé , “Benfotiamine Improves Dystrophic Pathology and Exercise Capacity in mdx Mice by Reducing Inflammation and Fibrosis,” Human Molecular Genetics 33, no. 15 (2024): 1339–1355. 38710523 10.1093/hmg/ddae066 PMC11262745

[125] M. Segatto , R. Szokoll , R. Fittipaldi , et al., “BETs Inhibition Attenuates Oxidative Stress and Preserves Muscle Integrity in Duchenne Muscular Dystrophy,” Nature Communications 11, no. 1 (2020): 6108. 10.1038/s41467-020-19839-x PMC7705749 33257646

